# Pharmacological and non-pharmacological interventions for irritability in autism spectrum disorder: a systematic review and meta-analysis with the GRADE assessment

**DOI:** 10.1186/s13229-024-00585-6

**Published:** 2024-01-23

**Authors:** Hangnyoung Choi, Jae Han Kim, Hee Sang Yang, Jong Yeob Kim, Samuele Cortese, Lee Smith, Ai Koyanagi, Elena Dragioti, Joaquim Radua, Paolo Fusar-Poli, Jae Il Shin, Keun-Ah Cheon, Marco Solmi

**Affiliations:** 1 Department of Child and Adolescent Psychiatry, Severance Hospital, Yonsei University College of Medicine, Yonsei-Ro 50, Seodaemun-Gu, Seoul, 03722 Republic of Korea; 2 Institute of Behavioral Science in Medicine, Yonsei University College of Medicine, Yonsei University Health System, Seoul, Republic of Korea; 3 Yonsei University College of Medicine, Severance Hospital, Yonsei University Health System, Seoul, Republic of Korea; 4 Centre for Innovation in Mental Health, School of Psychology, Faculty of Environmental and Life Sciences, University of Southampton, Southampton, UK; 5 Clinical and Experimental Sciences (CNS and Psychiatry), Faculty of Medicine, University of Southampton, Southampton, UK; 6 Solent NHS Trust, Southampton, UK; 7 Hassenfeld Children’s Hospital at NYU Langone, New York University Child Study Center, New York City, NY USA; 8 Centre for Health, Performance and Wellbeing, Anglia Ruskin University, Cambridge, CB1 1PT UK; 9 Parc Sanitari Sant Joan de Deu, Sant Boi de Llobregat ES, Barcelona, Spain; 10 Research Laboratory Psychology of Patients, Families and Health Professionals, Department of Nursing, School of Health Sciences, University of Ioannina, Ioannina, Greece; 11 Pain and Rehabilitation Centre, and Department of Medical and Health Sciences, Linköping University SE, Linköping, Sweden; 12 Imaging Mood- and Anxiety-Related Disorders (IMARD) Group, Institut d’Investigacions Biomèdiques August Pi i Sunyer (IDIBAPS), Mental Health Research Networking Center (CIBERSAM), University of Barcelona, Barcelona, Spain; 13 Department of Psychosis Studies, King’s College London, London, UK; 14 Department of Brain and Behavioral Sciences, University of Pavia, Pavia, Italy; 15 Outreach and Support in South-London (OASIS) Service, South London and Maudlsey (SLaM) NHS Foundation Trust, London, UK; 16 Department of Psychiatry and Psychotherapy, Ludwig-Maximilian-University Munich, Munich, Germany; 17 Department of Pediatrics, Yonsei University College of Medicine, Yonsei-Ro 50, Seodaemun-Gu, Seoul, 03722 Republic of Korea; 18 Severance Children’s Hospital, Yonsei University Health System, Seoul, Republic of Korea; 19 Severance Underwood Meta-Research Center, Institute of Convergence Science, Yonsei University, Seoul, Republic of Korea; 20 Department of Psychiatry, University of Ottawa, Ottawa, ON Canada; 21 Regional Centre for the Treatment of Eating Disorders and On Track: The Champlain First Episode Psychosis Program, Department of Mental Health, The Ottawa Hospital, Ottawa, ON Canada; 22 Ottawa Hospital Research Institute (OHRI), Clinical Epidemiology Program, University of Ottawa, Ottawa, ON Canada; 23 Department of Child and Adolescent Psychiatry, Charité Universitätsmedizin, Berlin, Germany; 24 Samsung Advanced Institute for Health Sciences & Technology (SAIHST), Sungkyunkwan University, Samsung Medical Center, Seoul, Republic of Korea; 25 DiMePRe-J-Department of Precision and Rigenerative Medicine-Jonic Area, University of Bari “Aldo Moro”, Bari, Italy

**Keywords:** Autism spectrum disorder, Systematic review, Meta-analysis, Irritability, Randomized controlled trial

## Abstract

**Background:**

Numerous interventions for irritability in autism spectrum disorder (ASD) have been investigated. We aimed to appraise the magnitude of pharmacological and non-pharmacological interventions for irritability in ASD without any restrictions in terms of eligible interventions.

**Methods:**

We systematically searched PubMed/MEDLINE, Scopus, and Web of Science until April 15, 2023. We included randomized controlled trials (RCTs) with a parallel design that examined the efficacy of interventions for the treatment of irritability in patients of any age with ASD without any restrictions in terms of eligible interventions. We performed a random-effects meta-analysis by pooling effect sizes as Hedges’ g. We classified assessed interventions as follows: pharmacological monotherapy, risperidone plus adjuvant therapy versus risperidone monotherapy, non-pharmacological intervention, and dietary intervention. We utilized the Cochrane tool to evaluate the risk of bias in each study and the GRADE approach to assess the certainty of evidence for each meta-analyzed intervention.

**Results:**

Out of 5640 references, we identified 60 eligible articles with 45 different kinds of interventions, including 3531 participants, of which 80.9% were males (mean age [SD] = 8.79 [3.85]). For pharmacological monotherapy, risperidone (Hedges’ g − 0.857, 95% CI − 1.263 to − 0.451, certainty of evidence: high) and aripiprazole (Hedges’ g − 0.559, 95% CI − 0.767 to − 0.351, certainty of evidence: high) outperformed placebo. Among the non-pharmacological interventions, parent training (Hedges’ g − 0.893, 95% CI − 1.184 to − 0.602, certainty of evidence: moderate) showed a significant result. None of the meta-analyzed interventions yielded significant effects among risperidone + adjuvant therapy and dietary supplementation. However, several novel molecules in augmentation to risperidone outperformed risperidone monotherapy, yet from one RCT each.

**Limitations:**

First, various tools have been utilized to measure the irritability in ASD, which may contribute to the heterogeneity of the outcomes. Second, meta-analyses for each intervention included only a small number of studies and participants.

**Conclusions:**

Only risperidone, aripiprazole among pharmacological interventions, and parent training among non-pharmacological interventions can be recommended for irritability in ASD. As an augmentation to risperidone, several novel treatments show promising effects, but further RCTs are needed to replicate findings.

*Trial registration* PROSPERO, CRD42021243965.

**Supplementary Information:**

The online version contains supplementary material available at 10.1186/s13229-024-00585-6.

## Background

Autism spectrum disorder (ASD) is a heritable and heterogeneous neurodevelopmental disorder defined by two core symptoms of impairments in social communication and restricted/repetitive behavioral patterns [1]. According to the Centers for Disease Control, approximately 1 in 36 children are diagnosed with ASD in the USA [2]. Patients with ASD commonly express not only the aforementioned core symptoms but also symptoms of irritability [3], and multimodal studies have been conducted to understand the nature of irritability in this population. They have revealed that aberrant responses to frustrative non-reward and aberrant approach responses to threat might be associated with irritable behavior. Brain regions related to these potential mechanisms have also been identified [4, 5]. Furthermore, environmental factors such as a volatile upbringing have been suggested to be associated with an aggressive personality, leading to irritable behavior [4].

Since irritability in ASD imposes a heavy burden on both patients themselves and their caregivers, it is a significant issue and primary treatment target. The Food and Drug Administration (FDA) has approved risperidone and aripiprazole for the treatment of irritability in ASD. Due to their potential risk of metabolic and neurological adverse events, however, other low-risk medications have been investigated [6]. Also, augmentation strategies to risperidone have been tested against risperidone monotherapy. Non-pharmacological interventions, such as parent training, have also been examined because they have advantages in terms of tolerability and safety compared to pharmacological interventions. However, the results have been inconsistent, and the magnitude of their effects was unclear. Herein, a systematic review with meta-analyses was recently performed on the pharmacological interventions for irritability and emotional dysregulation in this population [7]. Although providing well-summarized evidence including reports on risk of bias, they did not evaluate the level of certainty associated with the evidence. Moreover, non-pharmacological interventions were also not addressed, which were also widely recognized as an important approach to managing these symptoms.

Given the aforementioned, we conducted a systematic review and meta-analysis of randomized controlled trials (RCTs) to appraise the magnitude of interventions for irritability in patients with ASD. We did not apply any restrictions in terms of eligible interventions to clearly compare the efficacies among the identified interventions, using the standardized metrics for the effect. Additionally, we employed the Grading of Recommendations, Assessment, Development, and Evaluations (GRADE) method to evaluate the level of certainty in the evidence pertaining to the identified interventions. This approach facilitated a thorough assessment, particularly for those not approved by the FDA.

## Methods

### Study protocol and pre-registration

This study was performed according to Preferred Reporting Items for Systematic Reviews and Meta-analyses guidelines (Additional file 1: Table S1, 2) [8]. The protocol was registered with PROSPERO (CRD42021243965) with amendments during the study and peer-review processes (Additional file 1: Table S3). The process of screening, data extraction, and methodological evaluation of included articles was independently done by two authors (JHK and HSY), and any discrepancy was solved by discussion between the other authors (HC).

### Search strategy and selection criteria

**Fig. 1:**
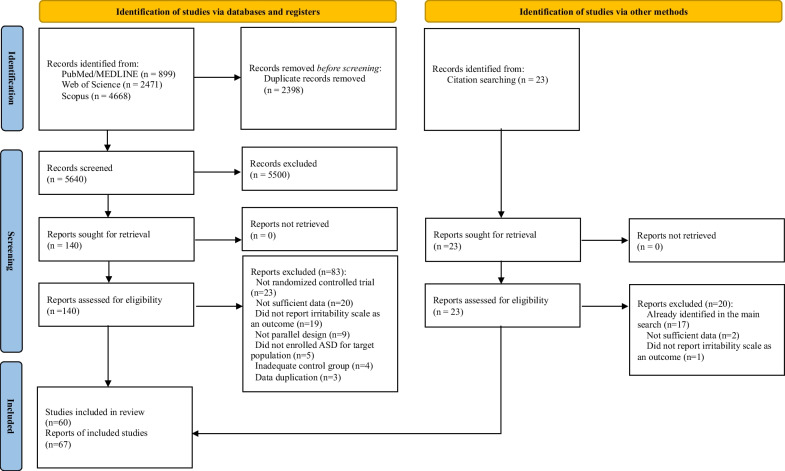
Study selection flow

We systematically searched PubMed/MEDLINE, Web of Science, and Scopus until April 15, 2023, without any language restrictions. Full search terms are listed in Additional file 1: Table S4. We screened titles, abstracts, and full texts and manually searched references of relevant studies to identify eligible articles (Fig. 1). We note that no tools for systematic review were employed throughout the screening process.

We included RCTs with a parallel design that examined the efficacy of interventions for the treatment of irritability in patients of any age with ASD without any restrictions in terms of eligible interventions. The ASD diagnosis was operationalized according to any version of the International Classification of Diseases, the Diagnostic and Statistical Manual of Mental Disorders, the Autism Diagnostic Interview, or the Autism Diagnostic Observation Schedule [1, 9–11]. We further included less rigorous diagnostic methods (such as a previous diagnosis by a professional) for ASD to widely identify interventions for irritability in this population. We included studies that reported irritability behavior scale as an outcome using validated methods such as Aberrant Behavior Checklist-Irritability (ABC-I), Developmental Behavior Checklist-Irritable (DBC-irritable), and Eyberg Child Behavior Inventory-Intensity (ECBI-intensity). Irritability was defined as excessive reactivity to negative emotional stimuli and described as having an affective component, anger, and a behavioral component, aggression [12].

We excluded studies that met the following exclusion criteria: trials other than parallel design (e.g., cross-over and discontinuation study), trials that did not include an adequate control group (e.g., placebo or inactive control), trials that did not enroll ASD patients for the target population, non-randomized controlled studies, trials that did not report irritability as an outcome, and trials that did not provide enough data needed for analysis. When multiple trials used the same sample, we prioritized the original trial since secondary trials mentioned the original one. The list of the excluded articles in full-text screening is presented in Additional file 1: Table S6, 7.

### Data extraction

From the eligible studies, we extracted the following data: the name of the first author; publication year; the country where the trial was done; details of the trial and patient characteristics (design of the trial, sample size, follow-up period, age range, mean age and standard deviation [SD], percentage of males); diagnostic criteria for ASD; measurement tool used to assess irritability; details of intervention in both treatment group and control group (type of intervention and its dose and duration); and results of the trial (effect size and corresponding 95% confidence interval [CI] or mean and SD of outcome measure at the baseline and end of treatment).

### Risk of bias and GRADE assessment

We evaluated the risk of bias for each eligible study using the updated Cochrane tool (RoB2) [13]. The following five domains were assessed: bias arising from the randomization process, bias due to deviations from intended interventions, bias due to missing outcome data, bias in the measurement of the outcome, and bias in the selection of the reported result. Each domain was judged as “low”, “some concerns”, or “high” risk of bias, and the overall risk of bias was determined according to these results.

We evaluated the certainty of the evidence using the GRADE approach for each meta-analysis [14]. The certainty of the evidence can be categorized as either ‘high,’ ‘moderate,’ ‘low,’ or ‘very low.’ Given that all individual studies we analyzed were RCTs, the initial assessment was designated as ‘high,’ which could then be downgraded to ‘moderate’ (1 step), ‘low’ (2 steps), or ‘very low’ (3 steps) based on various factors that reduce the certainty of the evidence, such as a high risk of bias. However, the downgrading of certainty can be counterbalanced by other factors that may increase the certainty, such as large effect size and dose–effect response gradient.

### Statistical analysis

We converted the reported effect sizes of interventions to standardized mean differences (Hedges’ g) and corresponding 95% CIs, which were then meta-analyzed. Considering the heterogeneous feature of ASD from a clinical aspect and the methodological diversities among included trials, meta-analysis was done under the random-effect models. The effect size of the intervention that was reported by a single trial, so meta-analysis was not available, was also converted into Hedges’ g with a corresponding 95% CI to enable the comparison of efficacy among the identified interventions. The magnitude of Hedges’ g can be interpreted as small (0.2–0.5), moderate (0.5–0.8), or large (> 0.8) according to Cohen’s convention [15].

When converting the raw effect sizes into Hedges’ g, the differences between intervention and control groups in changes in irritability scores were used. “Changes in irritability score” indicated the change of the score from the baseline to the end of treatment. For ease of interpretation, we transformed the estimates into negative values (negative effect size representing a reduction in irritability symptoms) since the purpose of the interventions is to reduce irritability in patients with ASD. When the effect size and 95% CI could not be calculated with given data (i.e., correlation coefficient [*r*] between baseline and end of treatment was not presented or could not be calculated), we used an imputed default value of 0.5 since it is the most conservative approach [16]. We employed the Knapp–Hartung adjustment in calculating a 95% CI for each combined estimate to minimize the risk of false positive results [17, 18]. To assess between-study heterogeneity, we performed Cochran’s Q test and calculated *I*^*2*^ statistics. The Q statistic represents the magnitude of statistical heterogeneity, and the *I*^*2*^ statistic represents the proportion of variance in the pooled effect size attributable to the heterogeneity [19]. We applied the restricted maximum likelihood estimator to evaluate the variance of heterogeneity, denoted as τ^2^ (tau square) [20]. We utilized Egger’s test and visual inspection of funnel plots to evaluate the publication bias [21].

We performed meta-regression analyses and subgroup analyses to assess potential moderating factors. Meta-regressions were done for publication year, sample size, mean age of the intervention group, and male percentage of the intervention group. Subgroup analyses were done for the overall risk of bias (measured by RoB2) and measurement tool for irritability. We conducted meta-regressions when at least four estimates were available. All statistical tests were two-sided and statistical significance was claimed at *P* < 0.05, and all statistical analyses were performed by R version 4.3.0 and its packages.

## Results

### Study selection and study characteristics

From the systematic search, we identified 5640 candidate articles after removing duplicates, of which 57 were eligible after the screening process. We also found three eligible articles by citation screening (Fig. 1). Finally, 60 articles were included, with a total of 3531 participants (median 47 participants per trial, interquartile range 38–66, range 12–218), containing 45 different kinds of interventions. The age of included trials ranges from 2 to 43 (mean age [SD] = 8.79 [3.85]) and the overall male percentage was 80.9%. We classified the assessed interventions as follows: (1) pharmacological monotherapy versus placebo, (2) risperidone + adjuvant therapy versus risperidone, (3) non-pharmacological intervention versus placebo, and (4) dietary supplementation versus placebo (Table 1). The detailed characteristics of included studies and their references are displayed in Additional file 1: Table S5.

**Table 1:** Summarized evidence for the efficacy of interventions for irritability in autism spectrum disorder

Interventions	*k*	Intervention group, *N*	Control group, *N*	Placebo group	Meta-analysis		Heterogeneity			Egger's test^a^	RoB2^b^	GRADE	Meta-regression coefficient for mean age of intervention group
					Hedges’ *g* (95% CI)	*P*	Q	*I*^*2*^	*τ*^*2*^	*P*			
*Pharmacological monotherapy versus placebo*													
Risperidone	6	187	204	Placebo	− 0.857 (− 1.263 to − 0.451)	**0.0029**	11.50	56.5	0.088	0.919	3L, 2M, 1H	High	0.1117 (*P* = 0.4215)
Aripiprazole	5	257	241	Placebo	− 0.559 (− 0.767 to − 0.351)	**0.0017**	2.68	0.0	0	0.415	5 L	High	0.0111 (*P* = 0.9580)
Lurasidone	2	99	98	Placebo	− 1.076 (− 3.884 to 1.732)	0.1289	2.09	52.2	0.051	NA	2 L	Moderate	
Anti-epileptic drug	3	39	33	Placebo	− 0.196 (− 1.219 to 0.828)	0.4970	1.98	0	0.008	0.824	2L, 1M	Low	
Valproate	2	29	23	Placebo	− 0.255 (− 5.127 to 4.619)	0.6270	1.83	45.3	0.133	NA	1L, 1M	Low	
Levetiracetam	1	10	10	Placebo	− 0.051 (− 0.927 to 0.825)	0.9092					L		
Balovaptan	1	86	81	Placebo	0.215 (− 0.089 to 0.519)	0.1654					H		
Amantadine hydrochloride	1	19	19	Placebo	− 0.609 (− 1.260 to 0.042)	0.0666					M		
Guanfacine	1	30	32	Placebo	− 0.481 (− 0.987 to 0.025)	0.0623					L		
Arbaclofen	1	61	69	Placebo	− 0.332 (− 0.679 to 0.015)	0.0607					L		
Mecamylamine	1	10	8	Placebo	− 0.180 (− 1.111 to 0.751)	0.7047					M		
Bumetanide	1	37	37	Placebo	− 0.135 (− 0.592 to 0.322)	0.5623					M		
*Risperidone + adjuvant therapy versus risperidone*													
Risperidone + dietary supplementation	5	111	109	Risperidone + placebo	− 0.490 (− 1.045 to 0.066)	0.0707	8.45	52.6	0.107	0.8799	3L, 2M	Very low	0.0025 (*P* = 0.9924)
Risperidone + N-acetylcysteine	2	37	34	Risperidone + placebo	− 0.677 (− 5.414 to 4.060)	0.3204	2.27	55.9	0.156	NA	1L, 1M	Very low	
Risperidone + sulforaphane	1	30	30	Risperidone + placebo	− 0.882 (− 1.411 to − 0.353)	**0.0011**					L		
Risperidone + L-Carnosine	1	21	21	Risperidone + placebo	− 0.198 (− 0.804 to 0.408)	0.5217					M		
Risperidone + *Ginkgo biloba*	1	23	24	Risperidone + placebo	− 0.028 (− 0.600 to 0.544)	0.9236					L		
Risperidone + Topiramate	1	20	20	Risperidone + placebo	− 1.983 (− 2.740 to − 1.226)	**< 0.0001**					L		
Risperidone + Pentoxifylline	1	20	20	Risperidone + placebo	− 1.785 (− 2.518 to − 1.052)	**< 0.0001**					L		
Risperidone + Memantine	1	20	20	Risperidone + placebo	− 1.534 (− 2.240 to − 0.828)	**< 0.0001**					L		
Risperidone + Celecoxib	1	20	20	Risperidone + placebo	− 1.276 (− 1.956 to − 0.596)	**0.0002**					L		
Risperidone + Minocycline	1	23	23	Risperidone + placebo	− 0.911 (− 1.519 to − 0.303)	**0.0033**					M		
Risperidone + Simvastatin	1	33	33	Risperidone + placebo	− 0.876 (− 1.382 to − 0.370)	**0.0007**					L		
Risperidone + Palmitoylethanolamide	1	31	31	Risperidone + placebo	− 0.873 (− 1.394 to − 0.352)	**0.001**					L		
Risperidone + Galantamine	1	20	20	Risperidone + placebo	− 0.775 (− 1.418 to − 0.132)	**0.018**					M		
Risperidone + Pioglitazone	1	20	20	Risperidone + placebo	− 0.770 (− 1.413 to − 0.127)	**0.019**					M		
Risperidone + Amantadine	1	20	19	Risperidone + placebo	− 0.747 (− 1.396 to − 0.098)	**0.024**					L		
Risperidone + Prednisolone	1	13	13	Risperidone + placebo	− 0.639 (− 1.427 to 0.149)	0.1119					L		
Risperidone + Pivotal Response Treatment	1	17	17	Risperidone	− 0.583 (− 1.269 to 0.103)	0.0958					H		
Risperidone + Propentofylline	1	24	24	Risperidone + placebo	− 0.562 (− 1.138 to 0.014)	0.0559					L		
Risperidone + Pregnenolone	1	30	29	Risperidone + placebo	− 0.503 (− 1.020 to 0.014)	0.0567					L		
Risperidone + Riluzole	1	20	20	Risperidone + placebo	− 0.496 (− 1.125 to 0.133)	0.1223					M		
Risperidone + Baclofen	1	29	29	Risperidone + placebo	− 0.432 (− 0.953 to 0.089)	0.1044					L		
Risperidone + Resveratrol	1	31	31	Risperidone + placebo	− 0.286 (− 0.786 to 0.214)	0.2620					L		
*Non-pharmacological intervention versus placebo*													
Parent training^c^	6	117	105	Waitlist control, placebo without waitlist	− 0.893 (− 1.184 to − 0.602)	**0.0005**	3.20	0.0	0	0.361	1M, 5H	Moderate	0.1793 (*P* = 0.0877)
Stepping Stone Triple P	2	41	39	Waitlist control	− 0.982 (− 1.448 to − 0.517)	**0.0237**	0.02	0.0	0	NA	2H	Moderate	
Therapeutic horseback riding	1	50	47	Barn activity	− 0.487 (− 0.891 to − 0.083)	**0.0181**					M		
Hyperbaric treatment (1.3 atm, 24% O_2_)	1	30	26	1.03 atm, 21% O_2_	− 0.445 (− 0.976 to 0.086)	0.1006					L		
Electro-acupuncture	1	30	25	Sham electro-acupuncture	− 0.109 (− 0.640 to 0.422)	0.6875					L		
*Dietary supplementation versus placebo*													
N-acetylcysteine	3	80	78	Placebo	− 0.151 (− 1.701 to 1.399)	0.7161	6.21	67.8	0.254	0.764	2L, 1M	Very low	
Polyunsaturated fatty acid	5	79	67	Placebo	− 0.235 (− 0.851 to 0.382)	0.3507	6.97	42.6	0.13	0.931	4L, 1M	Low	0.1690 (*P* = 0.3722)
Omega-3 fatty acid	4	72	61	Placebo	− 0.264 (− 1.127 to 0.599)	0.4021	6.83	56.1	0.178	0.969	3L, 1M	Very low	
Omega-3,6 fatty acid	1	7	6	Placebo	− 0.039 (− 1.129 to 1.051)	0.9441					L		
Vitamin D_3_	2	37	36	Placebo	− 0.298 (− 7.279 to 6.683)	0.6838	5.22	80.8	0.488	NA	1L, 1M	Very low	
Sulforaphane	1	26	14	Placebo	− 3.580 (− 4.599 to − 2.561)	**< 0.0001**					L		
Omega-3 fatty acid + vitamin D_3_	1	15	16	Placebo	− 0.604 (− 1.323 to 0.115)	0.0998					M		
Probiotics	1	18	17	Placebo	− 0.442 (− 1.112 to 0.228)	0.1962					L		
Folinic acid	1	23	25	Placebo	− 0.370 (− 0.940 to 0.200)	0.2036					L		

Bold indicate the statistical significance

*CI* confidence interval, *GRADE* Grading Of Recommendations, Assessment, Development, And Evaluations, *N* number of participants, *NA* not available, *k* number of estimates, *RoB2* risk of bias 2

^a^Egger's test might not have sufficient statistical power to identify publication bias when the number of studies is limited (i.e., when k is less than 10)

^b^RoB2 results of included trials are presented for each analysis (L = low, M = some concerns, H = high risk of bias)

^c^In our study, the term ‘parent training’ refers to parent training for maladaptive behaviors, as outlined in Bearrs’ taxonomy for parent training for ASD [22]

### Pharmacological monotherapy versus placebo

**Fig. 2:**
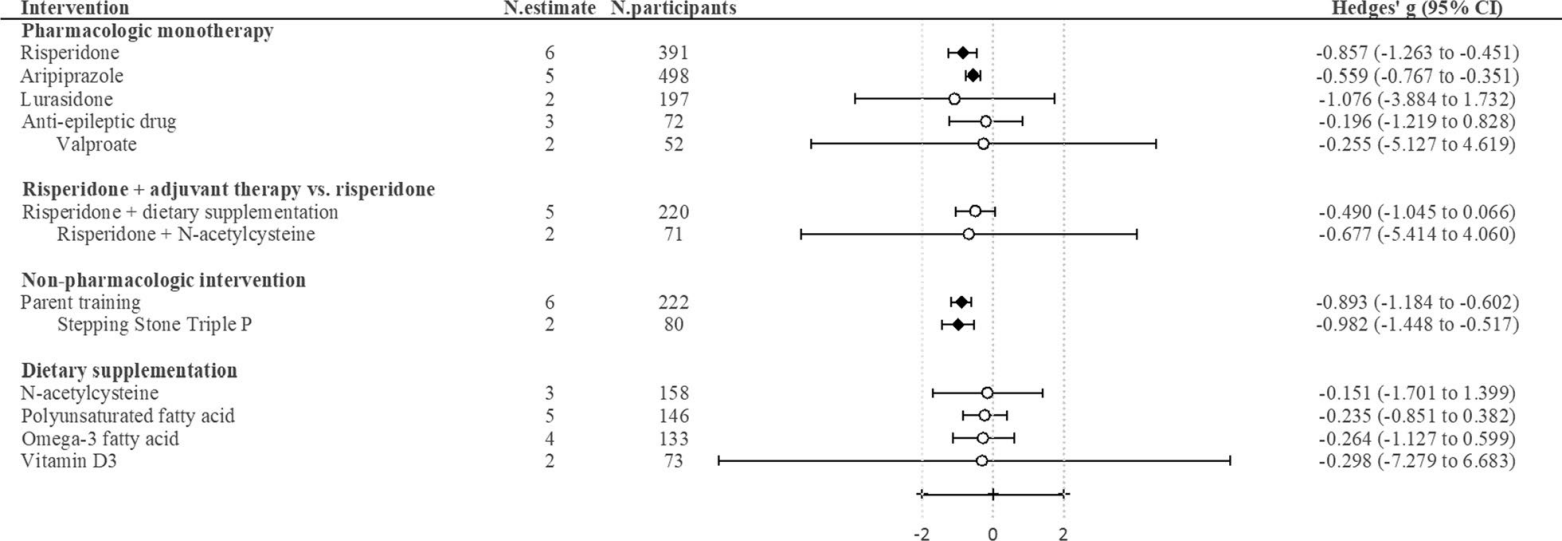
Summary of pooled estimates of meta-analyzed interventions for irritability in autism spectrum disorder. *CI* confidence interval, *N.estimate* number of estimates, *N.participants* number of participants. ^†^Black rhombus indicated statistical significance and white circles indicated nonsignificance

Meta-analyses of pharmacological monotherapy were available for risperidone, aripiprazole, lurasidone, anti-epileptic drugs, and valproate. Among these, risperidone (the number of estimates [*k*] = 6, Hedges’ g − 0.857, 95% CI − 1.263 to − 0.451, certainty of evidence: high) and aripiprazole (*k* = 5, Hedges’ g − 0.559, 95% CI − 0.767 to − 0.351, certainty of evidence: high) showed statistically significant effects on improvement in irritability score than placebo. However, statistical significance was not achieved for lurasidone (*k* = 2, Hedges’ g − 1.076, 95% CI − 3.884 to 1.732, certainty of evidence: moderate), anti-epileptic drugs (*k* = 3, Hedges’ g − 0.196, 95% CI − 1.219 to 0.828, certainty of evidence: low), and valproate (*k* = 2, Hedges’ g − 0.255, 95% CI − 5.127 to 4.619, certainty of evidence: low) (Table 1, Fig. 2).

### Risperidone + adjuvant therapy versus risperidone

Among the 60 eligible trials, 22 reported risperidone + adjuvant therapy versus risperidone. Dietary supplementation as adjuvant therapy was examined in five trials, while other candidate adjuvant therapies were investigated by a single trial. Meta-analysis on risperidone + dietary supplementation (*N*-acetylcysteine, sulforaphane, L-carnosine, and *Ginkgo biloba)* compared to risperidone did not reach statistical significance (*k* = 5, Hedges’ g − 0.490, 95% CI − 1.045 to 0.066, certainty of evidence: very low). The pooled estimate on risperidone + *N*-acetylcysteine vs. risperidone displayed a similar result (*k* = 2, Hedges’ g − 0.677, 95% CI − 5.414 to 4.060, certainty of evidence: very low). Several risperidone augmentation treatments were found to be superior to risperidone monotherapy in one single RCT each, including sulforaphane, topiramate, pentoxifylline, memantine, celecoxib, minocycline, simvastatin, palmitoylethanolamide, galantamine, pioglitazone, and amantadine, with effect sizes from moderate to large (Table 1, Fig. 2).

### Non-pharmacological intervention versus placebo

Regarding non-pharmacological interventions, meta-analysis was available for parent training and Stepping Stone Triple P. Identified forms of parenting training encompass Stepping Stone Triple P, Child-Directed Interaction Training, Parent Management Training (individual and workshop), and Parent training which adopted Research Units on Pediatric Psychopharmacology (RUPP) Parent Training Manual. These interventions could be classified as parent training for maladaptive behaviors, as outlined in Bearrs’ taxonomy for parent training for ASD [22]. Our meta-analysis showed that parent training had a better effect on reducing irritability scores than placebo (*k* = 6, Hedges’ g − 0.893, 95% CI − 1.184 to − 0.602, certainty of evidence: moderate). Stepping Stone Triple P also displayed a significant result, as shown by Hedges’ g of − 0.982 (95% CI − 1.448 to − 0.517, *k* = 2, certainty of evidence: moderate) (Table 1, Fig. 2).

### Dietary supplementation versus placebo

Regarding dietary supplementations, meta-analyses were available for *N*-acetylcysteine, polyunsaturated fatty acid, omega-3 fatty acid, and vitamin D3, none of which showed a better effect with statistical significance on reducing irritability scores than placebo. The certainty of evidence of these interventions ranges from ‘very low’ to ‘low.’ However, sulforaphane, which was reported by a single trial including 40 participants, reported a significant result (Hedges’ g − 3.580, 95% CI − 4.599 to − 2.561) (Table 1).

### Moderator analysis: meta-regression and subgroup analysis

We conducted meta-regression to assess potential moderators (publication year, sample size, the mean age of the intervention group, and male percentage of the intervention group) for each meta-analysis including more than three trials (Additional file 1: Table S8). None of them was found to have a significant moderating effect on the pooled effect size. Although the statistical significance was not obtained in our analysis (*P* = 0.0877), our results possibly suggested that parent training may be positively moderated by the mean age of the intervention group (meta-regression coefficient 0.1793, 95% CI − 0.0656 to 0.4243).

We performed subgroup analyses by the result of RoB2 (overall risk of bias) and measurement tools used to assess irritability (Additional file 1: Table S9). Notably, RoB2 was shown to be a moderating factor for polyunsaturated fatty acid (*P* = 0.0026), omega-3 fatty acid (*P* = 0.0024), and vitamin D3 (*P* = 0.0224). Specifically, the effect sizes of these interventions tended to be larger in trials with a higher risk of bias than in those with a lower risk of bias, which suggested that the effect of these interventions may be overestimated in trials with a higher risk of bias.

### Risk of bias assessment

**Fig. 3:**
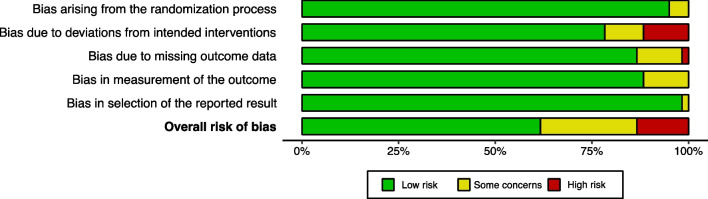
Summary plot of quality assessment (RoB2)

**Fig. 4:**
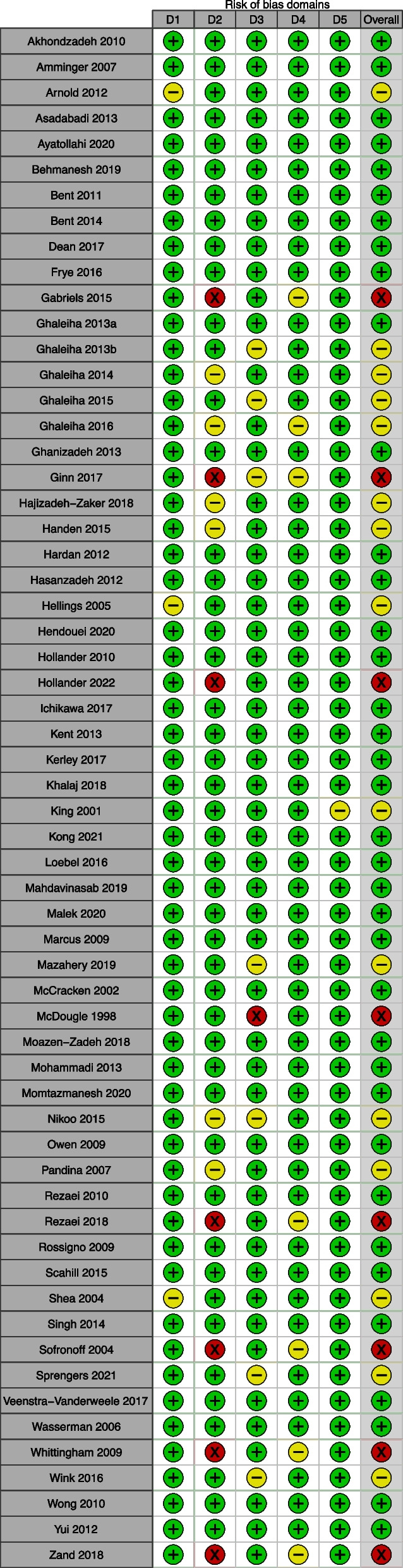
Quality assessment results for each domain of RoB2. ^†^Green plus = Low risk of bias, Yellow minus = Some concerns in risk of bias, Red cross = High risk of bias. ^‡^D1 = Bias arising from the randomization process, D2 = bias due to deviations from intended interventions, D3 = bias due to missing outcome data, D4 = bias in the measurement of the outcome, D5 = bias in the selection of the reported result

Out of 60 eligible articles, 37 (61.7%) showed a low overall risk of bias, 15 (25%) displayed some concerns about the overall risk of bias, and 8 (13.3%) were deemed to have a high overall risk of bias. The elevated overall risk of bias in 23 (38.5%) studies can be mainly attributed to biases arising from deviations from intended interventions, missing outcome data, and measurement of the outcome (Fig. 3). Among the eight studies that exhibited a high overall risk of bias, seven of which had deviations from intended interventions and one of which had a problem with missing outcome data (Fig. 4). Results of the risk of bias assessment and the reasons for judgment for each included study were presented in Additional file 1: Table S10.

## Discussion

The present study is a systematic review and meta-analysis that investigated pharmacological and non-pharmacological interventions for irritability in ASD without any restrictions in terms of eligible interventions. We also evaluated the certainty of evidence for meta-analyzed interventions to determine the robustness of the treatment effects observed across the included studies. Our study found 60 RCTs with a total of 3531 participants, containing 45 different kinds of interventions. Our meta-analysis found that risperidone, aripiprazole, and parent training were effective for the reduction of irritable symptoms in ASD compared to placebo or inactive control. Risperidone and aripiprazole demonstrated a high certainty of evidence according to the GRADE assessment, whereas parent training exhibited a moderate certainty of evidence. We also identified several promising molecules for augmentation to risperidone, yet from one RCT each.

For pharmacological interventions, only two showed a statistically significant effect on reducing irritability in ASD: risperidone had a large size effect (Hedges' g − 0.857, 95% CI − 1.263 to − 0.450) and aripiprazole had a moderate size (Hedges' g − 0.557, 95% CI − 0.766 to − 0.348) compared to placebo, while other monotherapy interventions such as lurasidone and anti-epileptic drugs reported doubtful results. Notably, both risperidone and aripiprazole displayed a high certainty of evidence. The efficacy of risperidone and aripiprazole can be explained by their biological mechanisms. The core brain circuitry mechanism related to irritability is regulated and mediated by neurotransmitters such as serotonin (5-HT), dopamine, noradrenaline, and gamma-aminobutyric acid. Therefore, medications capable of modulating these neurotransmitters have been widely used to treat irritability in various psychiatric disorders including ASD, psychosis, and oppositional-defiant disorder [23, 24]. Risperidone, a serotonin-dopamine antagonist, is closely related to this mechanism. Specifically, its antagonistic mechanisms on both the 5HT_2A_ receptor and dopamine D2 receptor have been reported to reduce irritable symptoms [25, 26]. Aripiprazole has distinctive receptor profiles compared to risperidone. It has not only a 5HT_2A_ receptor antagonistic effect but also partial agonistic effects on the D2 dopamine receptor and 5HT_1A_ serotonin receptor [27]. The effect of D2 receptor partial agonism can prevent hyperprolactinemia, thus improving compliance, especially in female patients [28]. Moreover, the 5HT_1A_ receptor has been found to be a serotonin receptor that is closely related to aggression, which may contribute to reducing irritability [29].

Some trials investigated the effectiveness of risperidone + adjuvant therapy compared to risperidone monotherapy. Notably, numerous drugs have been examined for adjuvant therapy, and trials on such non-psychotropic adjuvant therapy seemed to be based on the evidence that immune dysfunction in ASD was related to behavior problems [30, 31]. However, meta-analysis was available only for risperidone + dietary supplementation and did not report significant results, showing very low certainty of evidence. Although not meta-analyzed, on the other hand, some candidate adjuvant medications with only one trial (sulforaphane, topiramate, pentoxifylline, memantine, celecoxib, minocycline, simvastatin, palmitoylethanolamide, and amantadine) showed significant effects on reducing irritability in ASD with moderate to very large effect sizes. Considering some adjuvant medications for risperidone showed a potential to significantly reduce irritability score compared to risperidone monotherapy, further research on this topic should be warranted to replicate findings.

Concerning non-pharmacological interventions, parent training showed a promising effect in reducing irritability in ASD (Hedges’ g − 0.892, 95% CI − 1.184 to − 0.601) compared to inactive control with moderate certainty of evidence. Note that in this study, based on previously suggested classification of parent training for ASD [22], the term ‘parent training’ specifically refers to parent training aimed at addressing maladaptive behaviors (parent implementation), which emphasize skill development of parent and directly benefit the child. Our results are quite meaningful when considering the safety issues of pharmacotherapies [32]. Firstly, pharmacotherapies, including atypical antipsychotics, still have safety and tolerability issues. Even though a recent meta-analysis pointed out that antipsychotics are generally tolerable [33], numerous studies have reported adverse events related to pharmacotherapies such as extrapyramidal symptoms, somnolence, and weight gain, leading to poor compliance. Secondly, atypical antipsychotics, which were approved by the FDA for irritability, have an age limitation for use: risperidone is approved for those aged $\ge$ 5, and aripiprazole is for those aged $\ge$ 6. Therefore, non-pharmacological interventions such as parent training seem to be essential for children who have not yet reached the permitted age. Moreover, while not reaching statistical significance, our meta-regression analysis suggested that younger age may be associated with better outcomes of parent training (*k* = 4, meta-regression coefficient for the mean age of intervention group = 0.1793, *P* = 0.0877), which indicates early initiation of parent training may possibly be beneficial. We hypothesized that the nonsignificant result in this instance may be attributed to the limited number of studies and participants (four studies with a total of 222 participants), which consequently reduced the statistical power to detect the genuine effect. However, when considering early intervention is highly recommended for patients with ASD, the strategies that initiate early parent training before reaching the permitted age and combining parent training and pharmacological treatment when pharmacotherapies become available would be beneficial. Nevertheless, the notable efficacy of parent training should be interpreted in the context of limited evidence, as 5 out of 6 (83.3%) meta-analyzed estimates were associated with a high risk of bias, primarily due to a non-blind design of trials (open), which calls for future trials with double-blinded design.

While our study identified parent training, specifically the implementation aspect that emphasizes skill development of parent with the child as the primary beneficiary [22], it is important to note the existence of another form of parent training known as parent support (e.g., care coordination and psychoeducation) for addressing problematic behaviors [34–36]. However, parent support seemed to have lower efficacy compared to parent implementation. A previous RCT involving 180 children with ASD compared the efficacy of parent training (i.e., parent implementation) with parent education (i.e., parent support) and found that the former was superior to the latter in reducing problematic behaviors, including irritability [37].

For dietary supplementation, none of them exhibited a significant effect on the reduction of irritability in ASD except for sulforaphane. Sulforaphane was quite noticeable considering its large effect size even though only one trial was conducted. Notably, although not meta-analyzed, risperidone + sulforaphane also showed a better effect than risperidone monotherapy. Regarding *N*-acetylcysteine, whereas a previous meta-analysis reported that *N*-acetylcysteine may be efficacious [38], our analysis yielded nonsignificant results. Moreover, a pooled estimate on risperidone + *N*-acetylcysteine compared to risperidone monotherapy was also found to be nonsignificant. However, rather than interpreting these results as they are not helpful to alleviate irritability in ASD, the viewpoint that evidence is not yet enough to determine their effect and further studies are needed seems to be appropriate.

Due to the challenging nature of irritability in patients with ASD, it is a significant concern for both patients themselves and their caregivers. As a result, it has been a primary target of interventions including pharmacological agents such as risperidone and aripiprazole. However, due to the potential for adverse events, numerous studies have been done to extend the indication of existing medications for alleviating irritable symptoms in patients with ASD. A comprehensive systematic review with meta-analysis by Salazar de Pablo et al. investigated various pharmacological interventions in this context [7]. While re-affirming the efficacy of antipsychotics and identifying the potential of ADHD medications, the study did not cover the non-pharmacological interventions. Interestingly, our study revealed that the parent training demonstrated comparable efficacy to antipsychotics (risperidone and aripiprazole), suggesting that non-pharmacological interventions are as important as pharmacological interventions in managing irritability in patients with ASD. Notably, the strength of our study lies in its broad range of examined interventions, enabling the comparison of efficacy across different interventions using effect sizes of Hedge’s g.

In addition, our study went beyond the previous meta-analysis by utilizing the GRADE approach to evaluate the certainty of evidence for each meta-analyzed intervention. Since the GRADE approach addresses multiple dimensions of evidence such as risk of bias, imprecision, inconsistency, indirectness, and publication bias, it could provide a more comprehensive and nuanced perspective on the identified interventions compared to solely providing pooled effect size. For example, our study revealed parent training exhibited a comparable effect size to antipsychotics with moderate certainty of evidence. Interventions in other categories (risperidone + adjuvant therapy and dietary supplementation) yielded a certainty of evidence ranging from ‘very low’ to ‘low.’ These findings suggested that there is limited confidence in the reported efficacy of these interventions, indicating the need for further studies to establish robust evidence. By utilizing the GRADE approach to assess the certainty of evidence, our study enabled a more informed understanding of the efficacy of various interventions for irritability in patients with ASD. This approach guides future research and helps in making more evidence-based decisions in clinical practice.

## Limitations

The results of this study should be addressed in light of some limitations. First, various measurement tools for irritability were included such as ABC-I, DBC-irritable, and ECBI-Intensity, which may give the impression that outcomes may be influenced by the heterogeneity of measurements. However, out of 13 meta-analyses, only three included different measurements, and subgroup analyses for measurement tools showed that this was not a cause of between-study heterogeneity. Thus, the diversity of measurement tools seems not to have influenced the outcome considerably. Second, meta-analyses for each intervention included only a small number of studies and participants. However, this may be due to difficulties in conducting clinical trials for ASD, which is prevalent in children and adolescents. Indeed, most of the included studies targeted those aged under 19 years. Third, out of three interventions that were found to be effective for irritability in ASD, the results of risperidone and parent training may be influenced by the risk of bias. Indeed, three out of six trials of risperidone showed ‘some concerns’ or ‘high’ risk of bias, and all trials of parent training showed ‘some concerns’ or ‘high’ risk of bias. However, our subgroup analyses for risk of bias did not show major concerns. Fourth, the evidence of parent training primarily relied on RCTs without blinding. This is significant because open-label trial introduces the potential for bias, undermining the objectivity and credibility of the results, which suggested that the examined efficacy of parent training in this study may be overestimated.

## Conclusions

In conclusion, this study aggregated the evidence of interventions for irritability in ASD and present unified effect sizes. As a result, only risperidone and aripiprazole were pharmacological interventions with promising evidence, while other candidate medications had doubtful results. For non-pharmacological interventions, only parent training showed a significant effect on the reduction of irritability in ASD with moderate certainty of evidence. Several promising candidates as an augmentation to risperidone are found, for which findings should be replicated in additional RCTs.

### Supplementary Information

**Additional file 1**. Supplementary materials.

## Data Availability

The datasets used and/or analyzed during the current study are available from the corresponding authors (Jae Il Shin; shinji@yuhs.ac or Keun-Ah Cheon; kacheon@yuhs.ac) on reasonable request.

## Abbreviations

5-HT: Serotonin
ABC-I: Aberrant Behavior Checklist-Irritability
ASD: Autism spectrum disorder
CI: Confidence interval
DBC-irritable: Developmental Behavior Checklist-Irritable
ECBI-intensity: Eyberg Child Behavior Inventory-Intensity
FDA: Food and drug administration
GRADE: Grading of Recommendations, Assessment, Development, and Evaluations
k: The number of estimates
RCT: Randomized controlled trial
RoB2: Risk of bias 2
SD: Standard deviation
