# Supplementary material for: Pharmacological and non-pharmacological interventions for irritability in autism spectrum disorder: a systematic review and meta-analysis with the GRADE assessment
Source: Mol Autism. 2024 Jan 23;15:7. doi: 10.1186/s13229-024-00585-6 (PMC10807060; doi:10.1186/s13229-024-00585-6)
Supplement: Supplementary file 1 — Additional file 1. Supplementary materials. [file 13229_2024_585_MOESM1_ESM.pdf]

Supplementary materials

Pharmacological and non-pharmacological interventions for irritability in autism spectrum disorder: a systematic review and meta-analysis with the GRADE assessment

Hangnyoung Choi[1,2†], Jae Han Kim[3†], Hee Sang Yang[3†], Jong Yeob Kim[3], Samuele Cortese[4,5,6,7], Lee Smith[8], Ai Koyanagi[9], Elena Dragioti[10,11], Joaquim Radua[12], Paolo Fusar-Poli[13,14,15,16], Jae Il Shin[17,18,19*], Keun-Ah Cheon[1,2*], Marco Solmi[20,21,22,23]

Index

Table S1. PRISMA 2020 checklist 4

Table S2. PRISMA 2020 checklist for Abstracts 7

Table S3. Details on amendments of pre-registered protocol (CRD42021243965) 8

Table S4. Full search terms for each database (from inception to April 15, 2023) 9

Table S5. Characteristics of included trials 10

Table S6. The list of excluded articles in the full-text screening and reasons for exclusion (Identification of studies via databases and registers) 17

Table S7. The list of excluded articles in the full-text screening and reasons for exclusion (Identification of studies via other methods) 20

Table S8. Meta-regression analyses for (1) publication year, (2) sample size, (3) mean age of intervention group, and (4) male percentage of the intervention group. 21

1. Pharmacological monotherapy 21

2. Risperidone plus adjuvant therapy vs. risperidone 21

3. Non-pharmacological intervention 22

4. Dietary intervention 22

Table S9. Subgroup analyses for (1) RoB2 and (2) measurement tool for irritability 23

1. Pharmacological monotherapy 23

2. Risperidone plus adjuvant therapy vs. risperidone 24

3. Non-pharmacological intervention 24

4. Dietary intervention 25

Figure S1. Forest plot and funnel plot for each meta-analysis 26

I. Pharmacological intervention 26

1. Risperidone 26

2. Aripiprazole 26

3. Lurasidone 27

4. Anti-epileptic drugs 27

5. Valproate 28

II. Risperidone plus adjuvant therapy vs. risperidone 29

1. Risperidone + dietary supplementation vs. risperidone 29

2. Risperidone + N-acetylcysteine vs. risperidone 29

III. Non-pharmacological intervention 30

1. Parent training 30

2. Stepping Stones Triple P 30

IV. Dietary intervention 31

1. N-acetylcysteine 31

2. Polyunsaturated fatty acid 31

3. Omega-3 fatty acid 32

4. Vitamin D_3 32

Table S10. Details of risk of bias 2 assessment 33

Table S1. PRISMA 2020 checklist

Section and Topic	Item #	Checklist item	The location where item is reported
**TITLE**			
Title	1	Identify the report as a systematic review.	#1
**ABSTRACT**			
Abstract	2	See the PRISMA 2020 for Abstracts checklist.	Appendix p 7
**INTRODUCTION**			
Rationale	3	Describe the rationale for the review in the context of existing knowledge.	#4
Objectives	4	Provide an explicit statement of the objective(s) or question(s) the review addresses.	#4
**METHODS**			
Eligibility criteria	5	Specify the inclusion and exclusion criteria for the review and how studies were grouped for the syntheses.	#5
Information sources	6	Specify all databases, registers, websites, organisations, reference lists and other sources searched or consulted to identify studies. Specify the date when each source was last searched or consulted.	#5
Search strategy	7	Present the full search strategies for all databases, registers and websites, including any filters and limits used.	Appendix p 9
Selection process	8	Specify the methods used to decide whether a study met the inclusion criteria of the review, including how many reviewers screened each record and each report retrieved, whether they worked independently, and if applicable, details of automation tools used in the process.	#5, Figure 1
Data collection process	9	Specify the methods used to collect data from reports, including how many reviewers collected data from each report, whether they worked independently, any processes for obtaining or confirming data from study investigators, and if applicable, details of automation tools used in the process.	#5
Data items	10a	List and define all outcomes for which data were sought. Specify whether all results that were compatible with each outcome domain in each study were sought (e.g. for all measures, time points, analyses), and if not, the methods used to decide which results to collect.	#5
	10b	List and define all other variables for which data were sought (e.g. participant and intervention characteristics, funding sources). Describe any assumptions made about any missing or unclear information.	#5
Study risk of bias assessment	11	Specify the methods used to assess risk of bias in the included studies, including details of the tool(s) used, how many reviewers assessed each study and whether they worked independently, and if applicable, details of automation tools used in the process.	#6
Effect measures	12	Specify for each outcome the effect measure(s) (e.g. risk ratio, mean difference) used in the synthesis or presentation of results.	#6
Synthesis methods	13a	Describe the processes used to decide which studies were eligible for each synthesis (e.g. tabulating the study intervention characteristics and comparing against the planned groups for each synthesis (item #5)).	#6
	13b	Describe any methods required to prepare the data for presentation or synthesis, such as handling of missing summary statistics, or data conversions.	#6
	13c	Describe any methods used to tabulate or visually display results of individual studies and syntheses.	#6
	13d	Describe any methods used to synthesize results and provide a rationale for the choice(s). If meta-analysis was performed, describe the model(s), method(s) to identify the presence and extent of statistical heterogeneity, and software package(s) used.	#6
	13e	Describe any methods used to explore possible causes of heterogeneity among study results (e.g. subgroup analysis, meta-regression).	#6-7
	13f	Describe any sensitivity analyses conducted to assess robustness of the synthesized results.	#6-7

Section and Topic	Item #	Checklist item	The location where item is reported
Reporting bias assessment	14	Describe any methods used to assess risk of bias due to missing results in a synthesis (arising from reporting biases).	#6-7
Certainty assessment	15	Describe any methods used to assess certainty (or confidence) in the body of evidence for an outcome.	#6
**RESULTS**			
Study selection	16a	Describe the results of the search and selection process, from the number of records identified in the search to the number of studies included in the review, ideally using a flow diagram.	#8
	16b	Cite studies that might appear to meet the inclusion criteria, but which were excluded, and explain why they were excluded.	Appendix pp 17-20
Study characteristics	17	Cite each included study and present its characteristics.	Appendix pp 10-16
Risk of bias in studies	18	Present assessments of risk of bias for each included study.	Appendix pp 10-16, Figure 4
Results of individual studies	19	For all outcomes, present, for each study: (a) summary statistics for each group (where appropriate) and (b) an effect estimate and its precision (e.g. confidence/credible interval), ideally using structured tables or plots.	#8-9, Table 1, Figure 2, Appendix pp 26-32
Results of syntheses	20a	For each synthesis, briefly summarise the characteristics and risk of bias among contributing studies.	#8-9
	20b	Present results of all statistical syntheses conducted. If meta-analysis was done, present for each the summary estimate and its precision (e.g. confidence/credible interval) and measures of statistical heterogeneity. If comparing groups, describe the direction of the effect.	#8-9
	20c	Present results of all investigations of possible causes of heterogeneity among study results.	#9
	20d	Present results of all sensitivity analyses conducted to assess the robustness of the synthesized results.	#9
Reporting biases	21	Present assessments of risk of bias due to missing results (arising from reporting biases) for each synthesis assessed.	#9, Figure 3, 4
Certainty of evidence	22	Present assessments of certainty (or confidence) in the body of evidence for each outcome assessed.	#8-9, Table 1
**DISCUSSION**			
Discussion	23a	Provide a general interpretation of the results in the context of other evidence.	#10
	23b	Discuss any limitations of the evidence included in the review.	#12-13
	23c	Discuss any limitations of the review processes used.	#12-13
	23d	Discuss implications of the results for practice, policy, and future research.	#10-12
**OTHER INFORMATION**			

Section and Topic	Item #	Checklist item	The location where item is reported
Registration and protocol	24a	Provide registration information for the review, including register name and registration number, or state that the review was not registered.	#5
	24b	Indicate where the review protocol can be accessed, or state that a protocol was not prepared.	#5
	24c	Describe and explain any amendments to information provided at registration or in the protocol.	#5, Appendix p 8
Support	25	Describe sources of financial or non-financial support for the review, and the role of the funders or sponsors in the review.	#15
Competing interests	26	Declare any competing interests of review authors.	#15
Availability of data, code and other materials	27	Report which of the following are publicly available and where they can be found: template data collection forms; data extracted from included studies; data used for all analyses; analytic code; any other materials used in the review.	#15

Table S2. PRISMA 2020 checklist for Abstracts

Section and Topic	Item #	Checklist item	Reported (Yes/No)
**TITLE**			
Title	1	Identify the report as a systematic review.	Yes
**BACKGROUND**			
Objectives	2	Provide an explicit statement of the main objective(s) or question(s) the review addresses.	Yes
**METHODS**			
Eligibility criteria	3	Specify the inclusion and exclusion criteria for the review.	Yes
Information sources	4	Specify the information sources (e.g. databases, registers) used to identify studies and the date when each was last searched.	Yes
Risk of bias	5	Specify the methods used to assess risk of bias in the included studies.	Yes
Synthesis of results	6	Specify the methods used to present and synthesise results.	Yes
**RESULTS**			
Included studies	7	Give the total number of included studies and participants and summarise relevant characteristics of studies.	Yes
Synthesis of results	8	Present results for main outcomes, preferably indicating the number of included studies and participants for each. If meta-analysis was done, report the summary estimate and confidence/credible interval. If comparing groups, indicate the direction of the effect (i.e. which group is favoured).	Yes
**DISCUSSION**			
Limitations of evidence	9	Provide a brief summary of the limitations of the evidence included in the review (e.g. study risk of bias, inconsistency and imprecision).	Yes
Interpretation	10	Provide a general interpretation of the results and important implications.	Yes
**OTHER**			
Funding	11	Specify the primary source of funding for the review.	No (due to word count limitation)
Registration	12	Provide the register name and registration number.	Yes

Table S3. Details on amendments of pre-registered protocol (CRD42021243965)

Pre-registered protocol	Amendments	Reason
Searches<br>- Search dates - from inception to 21.03.20	From inception to April 15, 2023	During the submission process, we updated the search date, as the previous date had become outdated.
Types of study to be included<br>- Randomized controlled trials	Randomized controlled trials with parallel design	Our initial intention was to include a parallel design only, although this was not explicitly outlined in the protocol.
Participants/population<br>1) inclusion: participants of any age, with any type of autism spectrum disorder, Asperger's syndrome, pervasive developmental disorder not otherwise specified (PDD-NOS), autistic disorder, childhood disintegrative disorder, autistic disorder, and autism.<br>2) exclusion: participants with other neurodevelopmental disorders and mentally challenged adults (IQ < 70)	1) The ASD diagnosis was operationalized according to any version of the ICD, DSM, ADI, or ADOS. We further included less rigorous diagnostic methods (such as previous diagnosis by a professional)<br>2) No exclusion regarding patients' conditions	- We refined the operationalization of ASD diagnosis by incorporating criteria from the ICD, DSM, ADI, or ADOS to enhance sophistication. Concurrently, we also embraced less stringent diagnostic approaches to broadly identify interventions for irritability within this population.<br>- Due to the high comorbidity rate associated with autism spectrum disorder, it was not feasible to implement this exclusion criterion for the participants/population.
Main outcome(s)<br>- We consider any outcome measured by appropriate tool for irritability (e.g. Aberrant Behavior Checklist irritability subscale, the Clinical Global Impression-Improvement score, etc)	We included studies that reported irritability behavior scale as an outcome using validated methods such as Aberrant Behavior Checklist-Irritability (ABC-I), Developmental Behavior Checklist-Irritable (DBC-irritable), and Eyberg Child Behavior Inventory-Intensity (ECBI-intensity).	- 'DBC-irritable' and 'ECBI intensity' can also be used to assess patient's irritability.<br>- 'Clinical Global Impression-Improvement score' did not specifically measure patient's irritability.
Risk of bias (quality) assessment<br>- In accordance with the Cochrane Collaboration's tool for assessing the risk of bias, two independent reviewers will evaluate the risk of bias in included studies by considering the following factors: Bias arising from the randomization process, bias due to deviations from intended interventions, bias due to missing outcome data, bias in measurement of the outcome, and bias in selection of the reported result.	- We also assessed the certainty of the evidence using the GRADE approach for each meta-analysis.	- To provide more robust evidence on interventions for irritability in autism spectrum disorder.
Analysis of subgroups or subsets<br>- We plan subgroup analyses based on study design (open/single/double blind).	- We performed meta-regression analyses and subgroup analyses to assess potential moderating factors. Meta-regressions were done for publication year, sample size, mean age of the intervention group, and male percentage of the intervention group. Subgroup analyses were done for the overall risk of bias (measured by RoB2) and measurement tool for irritability.	- Subgroup analysis by study design was not feasible because study designs within meta-analyses were almost same. Consequently, we carried out additional meta-regression and subgroup analyses to furnish additional insights.

Table S4. Full search terms for each database (from inception to April 15, 2023)

**PubMed**: 899 articles were searched
("random*" OR "placebo" OR "control*" OR "RCT") AND ("autis*" OR "Asperg*" OR "pervasive developmental disorder") AND ("irritability" OR "agitation" OR "aggression" OR "temper tantrums" OR "self-injurious behavior" OR "problem behavior" OR "attention deficit and disruptive behavior disorder")
**Web of Science**: 2471 articles were searched
(random* OR placebo OR control* OR RCT) AND (autis* OR Asperg* OR (pervasive developmental disorder)) AND (irritability OR agitation OR aggression OR (temper tantrums) OR (self-injurious behavior) OR (problem behavior) OR (attention deficit and disruptive behavior disorder))
**Scopus**: 4668 articles were searched
(random* OR placebo OR control* OR RCT) AND (autis* OR Asperg* OR (pervasive developmental disorder)) AND (irritability OR agitation OR aggression OR (temper tantrums) OR (self-injurious behavior) OR (problem behavior) OR (attention deficit and disruptive behavior disorder))

Table S5. Characteristics of included trials

Author, year	Country	Design	Diagnostic criteria and collaborative tools	*N* at baseline	Age range (mean age ± SD)	% Male	Analyzed *N*	Intervention	Control	Period (weeks)	Dosage	Measurement tool for irritability	RoB2
**Akhondzadeh 2010**	Iran	double	DSM-IV-TR	40	4~12 (7.71 ± 2.22)	72.50%	40	Risperidone+Pentoxifylline	Risperidone +Placebo	10	Risperidone: 0.5~2 or 3mg/day, Pentoxifylline: 300~400 or 600mg/day	ABC-I	Low
**Amminger 2007**	NA	double	DSM-IV, ADI-R, ADOS	12	5~17 (10.4 ± 3.2)	81.90%	12	Omega-3 fatty acid and Vitamin (DHA, EPA, Vitamin E)	Placebo	6	DHA: 700mg/day, EPA: 840mg/day, Vitamin E: 7mg/day	ABC-I	Low
**Arnold 2012**	United States	double	DSM-IV, ADI-R	20	4~12 (7.4 ± 2.55)	85%	18	Mecamylamine	Placebo	14	0.5mg/day for first 6w, 2.5mg/day for next 2w, 5mg/day for last 6w	ABC-I	Some concerns
**Asadabadi 2013**	Iran	double	DSM-IV-TR, ADI-R	40	4~12 (7.55 ± 1.60)	62.50%	40	Risperidone+Celecoxib	Risperidone +Placebo	10	Risperidone: 0.5~2 or 3mg/day, Celecoxib: 100~200 or 300mg/day	ABC-I	Low
**Ayatollahi 2020**	Iran	double	DSM-5, ADI-R	59	11~17 (13.46 ± 2.00)	61%	59	Risperidone+Pregnenolone	Risperidone +Placebo	10	Risperidone: 0.5~2.5 or 3.5mg/day, Pregnenolone: 200mg/day	ABC-I	Low
**Behmanesh 2019**	Iran	double	DSM-5, ADI-R	48	4~11 (7.07 ± 2.13)	75%	48	Risperidone+Propentofylline	Risperidone +Placebo	10	Risperidone: 0.5~1 or 2mg/day, Propentofylline: 600 or 900mg/day	ABC-I	Low
**Bent 2011**	United States	double	DSM-IV TR, ADOS, SCQ	25	3~8 (5.83 ± 1.67)	NA	25	Omega-3 fatty acid (DHA, EPA)	Placebo	12	DHA: 460mg/day, EPA: 700mg/day	ABC-I	Low
**Bent 2014**	United States	double	Diagnosis by a professional, SCQ	57	5~8 (7.22 ± 1.06)	87.70%	57	Omega-3 fatty acid (DHA, EPA)	Placebo	6	DHA: 460mg/day, EPA: 700mg/day	ABC-I	Low
**Dean 2017**	Australia	double	DSM-IV-TR	98	3~9 (6.38 ± 1.9)	80.60%	98	N-acetylcysteine	Placebo	24	500mg/day	DBC-Irritable item	Low
**Frye 2016**	United States	double	DSM-5, ADI-R, ADOS	48	3~14 (7.37 ± 3.17)	79.20%	48	Folinic acid	Placebo	12	2mg/kg/day~50mg/day	ABC-I	Low
**Gabriels 2015**	United States	open	Previous diagnosis, ADOS or ADOS-2	116	6~16 (10.2 ± 3.0)	87%	97	Therapeutic Horseback Riding	Barn activity	10	NA	ABC-I	High
**Ghaleiha 2013a**	Iran	double	DSM-IV-TR, ADI-R	40	4~12 (7.70 ± 1.58)	57.50%	40	Risperidone+Memantine	Risperidone +Placebo	10	Risperidone: 0.5~2 or 3mg/day, Memantine: 5~15 or 20mg/day	ABC-I	Low
**Ghaleiha 2013b**	Iran	double	DSM-IV-TR, ADI-R	40	5~12 (8.00 ± 2.02)	82.50%	40	Risperidone+Riluzole	Risperidone +Placebo	10	Risperidone: 0.5~2 or 3mg/day, Riluzole: 25~50 or 100mg/day	ABC-I	Some concerns
**Ghaleiha 2014**	Iran	double	DSM-IV-TR, ADI-R	40	4~12 (6.38 ± 1.70)	87.50%	40	Risperidone+Galantamine	Risperidone +Placebo	10	Risperidone: 0.5~1 or 2mg/day, Galantamine: 2~12, 16, 20, or 24mg/day	ABC-I	Some concerns
**Ghaleiha 2015**	Iran	double	DSM-IV-TR, ADI-R	40	4~12 (6.58 ± 1.94)	80%	40	Risperidone+Pioglitazone	Risperidone +Placebo	10	Risperidone: 0.5~1 or 2mg/day, Pioglitazone: 30mg/day	ABC-I	Some concerns

**Ghaleiha 2016**	Iran	double	DSM-IV-TR, ADI-R	46	4~12 (7.79 ± 2.54)	76%	46	Risperidone+Minocycline	Risperidone +Placebo	10	Risperidone: 0.5~1 or 2mg/day, Minocycline: 100mg/day	ABC-I	Some concerns
**Ghanizadeh 2013**	Iran	double	DSM-IV-TR, ADI-R	40	3.5~16 (8.35 ± 2.77)	62.50%	31	Risperidone+N-acetylcysteine	Risperidone +Placebo	8	Risperidone: 0.5~2 or 3mg/day, N-acetylcysteine: 1200mg/day	ABC-I	Low
**Ginn 2017**	United States	open	Previous diagnosis by a healthcare professional, CARS-2	30	3~7 (4.72 ± 1.28)	80%	30	Child-Directed Interaction Training	Waitlist control	10	NA	ECBI intensity	High
**Hajizadeh-Zaker 2018**	Iran	double	DSM-5, ADI-R	42	4~12 (8.07 ± 2.06)	83.30%	42	Risperidone+L-carnosine	Risperidone +Placebo	10	Risperidone: 0.5~1 or 2mg/day, L-carnosine: 800mg/day	ABC-I	Some concerns
**Handen 2015**	United States	double	DSM-IV-TR, ADI-R	128	5~14.11 (8.1 ± 2.05)	85.20%	99	Parent Training + Placebo	Placebo	10	NA	ABC-I	Some concerns
**Hardan 2012**	United States	double	DSM-IV-TR, ADI-R, ADOS, expert clinical evaluation	29	3~12 (7.10 ± 2.15)	93.10%	29	N-acetylcysteine	Placebo	12	900mg/day for first 4w, 900mg bid for the next 4w, 900mg tid for the last 4w	ABC-I	Low
**Hasanzadeh 2012**	Iran	double	DSM-IV-TR, ADI-R	47	4~12 (6.42 ± 2.2)	83%	47	Risperidone+*Ginkgo biloba*	Risperidone +Placebo	10	Risperidone: 0.5~2 or 3mg/day, *Ginkgo biloba*: 80 or 120mg/day	ABC-I	Low
**Hellings 2005**	United States	double	DSM-IV, ADI-R, ADOS	36	6~20 (11.2 ± 6.06)	72.20%	25	Valproate	Placebo	8	20mg/kg/day	ABC-I	Some concerns
**Hendouei 2020**	Iran	double	DSM-5, ADI-R	62	4~12 (7.95 ± 2.00)	80.60%	62	Risperidone+Resveratrol	Risperidone +Placebo	10	Risperidone: 0.5~1 or 2mg/day, Resveratrol: 500mg/day	ABC-I	Low
**Hollander 2010**	United States	double	DSM-IV-TR, ADI-R, ADOS-G	27	4.85~14.92 (9.46 ± 2.65)	83.60%	27	Valproate	Placebo	12	125mg/day for 1w, 250mg/day at the next week, and personalized therapeutic drug level for remaining period	ABC-I	Low
**Hollander 2022**	United States	double	DSM-5, ADOS-2	167	5~17 (12.1 ± 3.4)	83.20%	167	Balovaptan	Placebo	24	10mg/day	ABC-I	High
**Ichikawa 2017**	Japan	double	DSM-IV-TR	92	6~17 (10.1 ± 3.2)	81.50%	92	Aripiprazole	Placebo	8	1~15mg/day	ABC-I	Low
**Kent 2013**	United States	double	DSM-IV, ADI-R	96	5~17 (9 ± 3.1)	87.50%	96	Risperidone	Placebo	6	0.125mg/day for low-dose group, 1.25mg/day for high-dose group	ABC-I	Low
**Kerley 2017**	Ireland	double	DSM (version NA), ADOS, DISCO	38	<18 (7.37 ± 3.62)	86.80%	38	Vitamin D3	Placebo	20	2000IU/day	ABC-I	Low
**Khalaj 2018**	Iran	double	DSM-5, ADI-R	62	4~12 (7.13 ± 2.23)	75.80%	62	Risperidone+Palmitoylethanolamide	Risperidone +Placebo	10	Risperidone: 0.5~1 or 2mg/day, Palmitoylethanolamide: 1200mg/day	ABC-I	Low

**King 2001**	United States	double	DSM-IV, ICD-10, ADI-R, ADOS	39	5~15 (7 ± NA)	87.20%	38	Amantadine hydrochloride	Placebo	4	2.5mg/kg/day for 1w, 5mg/kg/day for remaining 3w	ABC-I	Some concerns
**Kong 2021**	United States	double	DSM-IV-TR, ADOS-2, ADI-R	35	3~20 (NA ± NA)	74.3%	35	Probiotics (Lactobacillus plantarum PS128)	Placebo	28	6×10^{10} CFUs/day	ABC-I	Low
**Loebel 2016**	United States	double	DSM-IV-TR, ADI-R	148	6~17 (10.67 ± 3)	81.80%	148	Lurasidone	Placebo	6	20 or 60mg/day	ABC-I	Low
**Mahdavinasab 2019**	Iran	double	DSM-5, ADI-R	58	4~12 (7.97 ± 2.17)	79.30%	58	Risperidone+Baclofen	Risperidone +Placebo	10	Risperidone: 0.5~1 or 2mg/day, Baclofen: 0.6mg/kg/day	ABC-I	Low
**Malek 2020**	Iran	single	DSM-5, ADI-R	26	3~12 (6.08 ± 2.31)	96.20%	26	Risperidone+Prednisolone	Risperidone +Placebo	12	Risperidone: 0.5~1 or 2mg/day, Prednisolone: 1mg/kg/day	ABC-I	Low
**Marcus 2009**	United States	double	DSM-IV-TR, ADI-R	218	6~17 (9.68 ± 3.03)	89.40%	213	Aripiprazole	Placebo	8w	5, 10, or 15mg/day	ABC-I	Low
**Mazahery 2019**	New Zealand	double	DSM-5	73	2.5~8 (5.25 ± 1.36)	82.20%	73	Omega-3 fatty acid and/or Vitamin (DHA, Vitamin D3, or DHA+Vitamin D3)	Placebo	1 year	DHA: 722mg/day, Vitamin D3: 2000IU/day	ABC-I	Some concerns
**McCracken 2002**	United States	double	DSM-IV	101	5~17 (8.8 ± 2.7)	81.20%	101	Risperidone	Placebo	8	0.5~2.5mg/day for children with 20~45kg, 0.25~mg/day for those under 20kg	ABC-I	Low
**McDougle 1998**	United States	double	DSM-IV, ADI-R, ADOS	31	18~43 (28.1 ± 7.3)	71.00%	30	Risperidone	Placebo	12	1~10mg/day	SIB-Q	High
**Moazen-Zadeh 2018**	Iran	double	DSM-IV-TR, ADI-R	66	4~12 (7.34 ± 2.54)	80.30%	66	Risperidone+Simvastatin	Risperidone +Placebo	10	Risperidone: 0.5~1 or 2mg/day, Simvastatin: 20 or 40mg/day	ABC-I	Low
**Mohammadi 2013**	Iran	double	DSM-IV-TR, ADI-R	40	4~12 (6.75 ± 2.35)	82.50%	39	Risperidone+Amantadine	Risperidone +Placebo	10	Risperidone: 0.5~2mg/day, Amantadine: 100~150mg/day	ABC-I	Low
**Momtazmanes h 2020**	Iran	double	DSM-5, ADI-R	60	4~12 (7.27 ± 2.21)	66.70%	60	Risperidone+Sulforaphane	Risperidone +Placebo	10	Risperidone: 0.25 or 0.5 ~ 1, 2.5, or 3.5mg/day, Sulforaphane: 50 or 100 μmol/day	ABC-I	Low
**Nikoo 2015**	Iran	double	DSM-IV-TR, ADI-R	40	4~12 (7.55 ± 2.61)	82.50%	40	Risperidone+N-acetylcysteine	Risperidone +Placebo	10	Risperidone: 0.5~1 or 2mg/day, N-acetylcysteine: 600~900mg/day	ABC-I	Some concerns
**Owen 2009**	United States	double	DSM-IV-TR, ADI-R	98	6~17 (8.38 ± 2.9)	87.80%	95	Aripiprazole	Placebo	8	5, 10, or 15mg/day	ABC-I	Low
**Pandina 2007**	Canada	double	DSM-IV, CARS	55	5~12 (7.25 ± 2.21)	78.20%	52	Risperidone	Placebo	8	0.01~0.06mg/kg/day	ABC-I	Some concerns
**Rezaei 2010**	Iran	double	DSM-IV-TR, ADI-R	40	3~12 (8.01 ± 1.89)	67.50%	40	Risperidone+Topiramate	Risperidone +Placebo	8	Risperidone: 0.5~2 or 3mg/day, Topiramate: 100 or 200mg/day	ABC-I	Low

**Rezaei 2018**	Iran	open	DSM-5	34	NA (12.24 ± 2.81)	65%	34	Risperidone+Pivotal Response Treatment	Risperidone	12	Risperidone: 0.5~2.5 or 3.5mg/day	ABC-I	High
**Rossigno 2009**	United States	double	DSM-IV, ADI-R, ADOS	62	2~7 (4.92 ± 1.22)	83.90%	56	Hyperbaric Treatment (1.3atm, 24% O2)	1.03atm, 21% O2	4	NA	ABC-I	Low
**Scahill 2015**	United States	double	DSM-IV, ADOS, SCQ	62	5~14 (8.5 ± 2.25)	85.50%	62	Guanfacine	Placebo	8	1~4mg/day	ABC-I	Low
**Shea 2004**	Canada	double	DSM-IV, CARS	79	5~12 (7.45 ± 2.3)	77.20%	77	Risperidone	Placebo	8	0.01~0.06mg/kg/day	ABC-I	Some concerns
**Singh 2014**	United States	double	DSM-IV, ADOS	44	13~27 (NA ± NA)	100%	40	Sulforaphane	Placebo	18	50, 100, or 150μmol/day	ABC-I	Low
**Sofronoff 2004**	Australia	open	Recent diagonosis by the consultant paediatrician	51	6~12 (9.33 ± NA)	NA	51	Parent Management Training (Individual, Workshop)	Waitlist control	4	NA	ECBI intensity	High
**Sprengers 2021**	Netherlan ds	double	DSM-IV-TR, ADOS-2	92	7~15 (10.5 ± 2.4)	68.4%	74	Bumetanide	Placebo	91 days	1.0mg bid	ABC-I	Some concerns
**Veenstra-Vanderweele 2017**	United States	double	DSM-IV-TR	150	5~21 (11.6 ± 4.6)	82.70%	130	Arbaclofen	Placebo	12	5mg bid for the first week, 10mg bid for the second week, 10mg tid for the third week, 15mg tid for the remaining (10mg tid is maximum for <12 years)	ABC-I	Low
**Wasserman 2006**	United States	double	DSM-IV, ADI-R, ADOS	20	5~17 (8.72 ± 3.16)	85%	20	Levetiracetam	Placebo	10	125mg/day for 4w, 250mg/day for the next 4w, +20~30mg/day for the last 2w	ABC-I	Low
**Whittingham 2009**	Australia	open	Primary diagnosis by a pediatrician, DSM-IV	59	2~9 (5.91 ± 1.90)	79.70%	59	Stepping Stones Triple P	Waitlist control	9	NA	ECBI intensity	High
**Wink 2016**	United States	double	DSM-IV, ADI-R	31	4~12 (7.89 ± 2.7)	77.40%	31	N-acetylcysteine	Placebo	12	Target dose: 60mg/kg/day	ABC-I	Some concerns
**Wong 2010**	Hong Kong	double	DSM-IV, ADI-R, ADOS	55	3~18 (9.25 ± 4.17)	85.50%	55	Electro-Acupuncture	Sham electro-acupuncture	4	NA	ABC-I	Low
**Yui 2012**	Japan	double	DSM-IV, ADI-R	13	6~28 (14.6 ± 6.0)	92.30%	13	Omega-3, 6 fatty acid (ARA, DHA)	Placebo	16	ARA: 240mg/day, DHA: 240mg/day	ABC-I	Low
**Zand 2018**	United States	open	DSM-5	21	2~12 (5.84 ± 2.92)	85.70%	21	Stepping Stones Triple P	Waitlist control	4	NA	ECBI intensity	High

Abbreviations: ABC-I=Aberrant Behavior Checklist-Irritability, ADI-R=Autism Diagnostic Interview-Revised, ADOS=Autism Diagnostic Observation Schedule, ARA=Arachidonic acid, bid=Twice a day, CARS=Childhood Autism Rating Scale, DBC=Developmental Behavior Checklist, DHA=Docosahexaenoic acid, DISCO=The Diagnostic Interview for Social and Communication Disorders , DSM=Diagnostic and Statistical Manual of Mental Disorders, ECBI=Eyberg Child Behavior Inventory, EPA=Eicosapentaenoic acid, N=Number of participants, NA=Not available, RoB2 =Risk of bias 2, SCQ=Social Communication Questionnaire, SD=Standard deviation, SIB-Q=Self-Injurious Behavior Questionnaire, tid=Three time a day, w=week

† Study that did not report the information on patients' age was excluded in calculation.

1. Akhondzadeh S, Fallah J, Mohammadi MR, et al. Double-blind placebo-controlled trial of pentoxifylline added to risperidone: effects on aberrant behavior in children with autism. *Prog Neuropsychopharmacol Biol Psychiatry* 2010; **34**(1): 32-6.
2. Amminger GP, Berger GE, Schäfer MR, Klier C, Friedrich MH, Feucht M. Omega-3 fatty acids supplementation in children with autism: a double-blind randomized, placebo-controlled pilot study. *Biol Psychiatry* 2007; **61**(4): 551-3.
3. Arnold LE, Aman MG, Hollway J, et al. Placebo-controlled pilot trial of mecamylamine for treatment of autism spectrum disorders. *J Child Adolesc Psychopharmacol* 2012; **22**(3): 198-205.
4. Asadabadi M, Mohammadi MR, Ghanizadeh A, et al. Celecoxib as adjunctive treatment to risperidone in children with autistic disorder: a randomized, double-blind, placebo-controlled trial. *Psychopharmacology (Berl)* 2013; **225**(1): 51-9.
5. Ayatollahi A, Bagheri S, Ashraf-Ganjouei A, Moradi K, Mohammadi MR, Akhondzadeh S. Does Pregnenolone Adjunct to Risperidone Ameliorate Irritable Behavior in Adolescents With Autism Spectrum Disorder: A Randomized, Double-Blind, Placebo-Controlled Clinical Trial? *Clin Neuropharmacol* 2020; **43**(5): 139-45.
6. Behmanesh H, Moghaddam HS, Mohammadi MR, Akhondzadeh S. Risperidone Combination Therapy With Propentofylline for Treatment of Irritability in Autism Spectrum Disorders: A Randomized, Double-Blind, Placebo-Controlled Clinical Trial. *Clin Neuropharmacol* 2019; **42**(6): 189-96.
7. Bent S, Bertoglio K, Ashwood P, Bostrom A, Hendren RL. A pilot randomized controlled trial of omega-3 fatty acids for autism spectrum disorder. *J Autism Dev Disord* 2011; **41**(5): 545-54.
8. Bent S, Hendren RL, Zandi T, et al. Internet-based, randomized, controlled trial of omega-3 fatty acids for hyperactivity in autism. *J Am Acad Child Adolesc Psychiatry* 2014; **53**(6): 658-66.
9. Dean OM, Gray KM, Villagonzalo KA, et al. A randomised, double blind, placebo-controlled trial of a fixed dose of N-acetyl cysteine in children with autistic disorder. *Aust N Z J Psychiatry* 2017; **51**(3): 241-9.
10. Frye RE, Slattery J, Delhey L, et al. Folinic acid improves verbal communication in children with autism and language impairment: a randomized double-blind placebo-controlled trial. *Mol Psychiatry* 2018; **23**(2): 247-56.
11. Gabriels RL, Pan Z, Dechant B, Agnew JA, Brim N, Mesibov G. Randomized Controlled Trial of Therapeutic Horseback Riding in Children and Adolescents With Autism Spectrum Disorder. *J Am Acad Child Adolesc Psychiatry* 2015; **54**(7): 541-9.
12. Ghaleiha A, Alikhani R, Kazemi MR, et al. Minocycline as Adjunctive Treatment to Risperidone in Children with Autistic Disorder: A Randomized, Double-Blind Placebo-Controlled Trial. *J Child Adolesc Psychopharmacol* 2016; **26**(9): 784-91.
13. Ghaleiha A, Asadabadi M, Mohammadi MR, et al. Memantine as adjunctive treatment to risperidone in children with autistic disorder: a randomized, double-blind, placebo-controlled trial. *Int J Neuropsychopharmacol* 2013; **16**(4): 783-9.
14. Ghaleiha A, Ghyasvand M, Mohammadi MR, et al. Galantamine efficacy and tolerability as an augmentative therapy in autistic children: A randomized, double-blind, placebo-controlled trial. *J Psychopharmacol* 2014; **28**(7): 677-85.
15. Ghaleiha A, Mohammadi E, Mohammadi MR, et al. Riluzole as an adjunctive therapy to risperidone for the treatment of irritability in children with autistic disorder: a double-blind, placebo-controlled, randomized trial. *Paediatr Drugs* 2013; **15**(6): 505-14.
16. Ghaleiha A, Rasa SM, Nikoo M, Farokhnia M, Mohammadi MR, Akhondzadeh S. A pilot double-blind placebo-controlled trial of pioglitazone as adjunctive treatment to risperidone: Effects on aberrant behavior in children with autism. *Psychiatry Res* 2015; **229**(1-2): 181-7.
17. Ghanizadeh A, Moghimi-Sarani E. A randomized double blind placebo controlled clinical trial of N-Acetylcysteine added to risperidone for treating autistic disorders. *BMC Psychiatry* 2013; **13**: 196.
18. Ginn NC, Clionsky LN, Eyberg SM, Warner-Metzger C, Abner JP. Child-Directed Interaction Training for Young Children With Autism Spectrum Disorders: Parent and Child Outcomes. *J Clin Child Adolesc Psychol* 2017; **46**(1): 101-9.
19. Hajizadeh-Zaker R, Ghajar A, Mesgarpour B, Afarideh M, Mohammadi MR, Akhondzadeh S. l-Carnosine As an Adjunctive Therapy to Risperidone in Children with Autistic Disorder: A Randomized, Double-Blind, Placebo-Controlled Trial. *J Child Adolesc Psychopharmacol* 2018; **28**(1): 74-81.
20. Handen BL, Aman MG, Arnold LE, et al. Atomoxetine, Parent Training, and Their Combination in Children With Autism Spectrum Disorder and Attention-Deficit/Hyperactivity Disorder. *J Am Acad Child Adolesc Psychiatry* 2015; **54**(11): 905-15.
21. Hardan AY, Fung LK, Libove RA, et al. A randomized controlled pilot trial of oral N-acetylcysteine in children with autism. *Biol Psychiatry* 2012; **71**(11): 956-61.
22. Hasanzadeh E, Mohammadi MR, Ghanizadeh A, et al. A double-blind placebo controlled trial of Ginkgo biloba added to risperidone in patients with autistic disorders. *Child Psychiatry Hum Dev* 2012; **43**(5): 674-82.
23. Hellings JA, Weckbaugh M, Nickel EJ, et al. A double-blind, placebo-controlled study of valproate for aggression in youth with pervasive developmental disorders. *J Child Adolesc Psychopharmacol* 2005; **15**(4): 682-92.

24. Hendouei F, Sanjari Moghaddam H, Mohammadi MR, Taslimi N, Rezaei F, Akhondzadeh S. Resveratrol as adjunctive therapy in treatment of irritability in children with autism: A double-blind and placebo-controlled randomized trial. *J Clin Pharm Ther* 2020; **45**(2): 324-34.

25. Hollander E, Chaplin W, Soorya L, et al. Divalproex sodium vs placebo for the treatment of irritability in children and adolescents with autism spectrum disorders. *Neuropsychopharmacology* 2010; **35**(4): 990-8.

26. Hollander E, Jacob S, Jou R, et al. Balovaptan vs Placebo for Social Communication in Childhood Autism Spectrum Disorder: A Randomized Clinical Trial. *JAMA Psychiatry*. 2022; 79(8): 760-769.

27. Ichikawa H, Mikami K, Okada T, et al. Aripiprazole in the Treatment of Irritability in Children and Adolescents with Autism Spectrum Disorder in Japan: A Randomized, Double-blind, Placebo-controlled Study. *Child Psychiatry Hum Dev* 2017; **48**(5): 796-806.

28. Kent JM, Kushner S, Ning X, et al. Risperidone dosing in children and adolescents with autistic disorder: a double-blind, placebo-controlled study. *J Autism Dev Disord* 2013; **43**(8): 1773-83.

29. Kerley CP, Power C, Gallagher L, Coghlan D. Lack of effect of vitamin D(3) supplementation in autism: a 20-week, placebo-controlled RCT. *Arch Dis Child* 2017; **102**(11): 1030-6.

30. Khalaj M, Saghazadeh A, Shirazi E, et al. Palmitoylethanolamide as adjunctive therapy for autism: Efficacy and safety results from a randomized controlled trial. *J Psychiatr Res* 2018; **103**: 104-11.

31. King BH, Wright DM, Handen BL, et al. Double-blind, placebo-controlled study of amantadine hydrochloride in the treatment of children with autistic disorder. *J Am Acad Child Adolesc Psychiatry* 2001; **40**(6): 658-65.

32. Kong XJ, Liu J, Liu K, et al. Probiotic and Oxytocin Combination Therapy in Patients with Autism Spectrum Disorder: A Randomized, Double-Blinded, Placebo-Controlled Pilot Trial. *Nutrients*. 2021; 13(5): 1552.

33. Loebel A, Brams M, Goldman RS, et al. Lurasidone for the Treatment of Irritability Associated with Autistic Disorder. *J Autism Dev Disord* 2016; **46**(4): 1153-63.

34. Mahdavinasab SM, Saghazadeh A, Motamed-Gorji N, et al. Baclofen as an adjuvant therapy for autism: a randomized, double-blind, placebo-controlled trial. *Eur Child Adolesc Psychiatry* 2019; **28**(12): 1619-28.

35. Malek M, Ashraf-Ganjouei A, Moradi K, Bagheri S, Mohammadi MR, Akhondzadeh S. Prednisolone as Adjunctive Treatment to Risperidone in Children With Regressive Type of Autism Spectrum Disorder: A Randomized, Placebo-Controlled Trial. *Clin Neuropharmacol* 2020; **43**(2): 39-45.

36. Marcus RN, Owen R, Kamen L, et al. A placebo-controlled, fixed-dose study of aripiprazole in children and adolescents with irritability associated with autistic disorder. *J Am Acad Child Adolesc Psychiatry* 2009; **48**(11): 1110-9.

37. Mazahery H, Conlon CA, Beck KL, et al. A randomised controlled trial of vitamin D and omega-3 long chain polyunsaturated fatty acids in the treatment of irritability and hyperactivity among children with autism spectrum disorder. *J Steroid Biochem Mol Biol* 2019; **187**: 9-16.

38. McCracken JT, McGough J, Shah B, et al. Risperidone in children with autism and serious behavioral problems. *N Engl J Med* 2002; **347**(5): 314-21.

39. McDougle CJ, Holmes JP, Carlson DC, Pelton GH, Cohen DJ, Price LH. A double-blind, placebo-controlled study of risperidone in adults with autistic disorder and other pervasive developmental disorders. *Arch Gen Psychiatry* 1998; **55**(7): 633-41.

40. Moazen-Zadeh E, Shirzad F, Karkhaneh-Yousefi MA, Khezri R, Mohammadi MR, Akhondzadeh S. Simvastatin as an Adjunctive Therapy to Risperidone in Treatment of Autism: A Randomized, Double-Blind, Placebo-Controlled Clinical Trial. *J Child Adolesc Psychopharmacol* 2018; **28**(1): 82-9.

41. Mohammadi MR, Yadegari N, Hassanzadeh E, et al. Double-blind, placebo-controlled trial of risperidone plus amantadine in children with autism: a 10-week randomized study. *Clin Neuropharmacol* 2013; **36**(6): 179-84.

42. Momtazmanesh S, Amirimoghaddam-Yazdi Z, Moghaddam HS, Mohammadi MR, Akhondzadeh S. Sulforaphane as an adjunctive treatment for irritability in children with autism spectrum disorder: A randomized, double-blind, placebo-controlled clinical trial. *Psychiatry Clin Neurosci* 2020; **74**(7): 398-405.

43. Nikoo M, Radnia H, Farokhnia M, Mohammadi MR, Akhondzadeh S. N-acetylcysteine as an adjunctive therapy to risperidone for treatment of irritability in autism: a randomized, double-blind, placebo-controlled clinical trial of efficacy and safety. *Clin Neuropharmacol* 2015; **38**(1): 11-7.

44. Owen R, Sikich L, Marcus RN, et al. Aripiprazole in the treatment of irritability in children and adolescents with autistic disorder. *Pediatrics* 2009; **124**(6): 1533-40.

45. Pandina GJ, Bossie CA, Youssef E, Zhu Y, Dunbar F. Risperidone improves behavioral symptoms in children with autism in a randomized, double-blind, placebo-controlled trial. *J Autism Dev Disord* 2007; **37**(2): 367-73.

46. Rezaei M, Moradi A, Tehrani-Doost M, Hassanabadi H, Khosroabadi R. Effects of Combining Medication and Pivotal Response Treatment on Aberrant Behavior in Children with Autism Spectrum Disorder. *Children (Basel)* 2018; **5**(2).

47. Rezaei V, Mohammadi MR, Ghanizadeh A, et al. Double-blind, placebo-controlled trial of risperidone plus topiramate in children with autistic disorder. *Prog Neuropsychopharmacol Biol Psychiatry* 2010; **34**(7): 1269-72.

48. Rossignol DA, Rossignol LW, Smith S, et al. Hyperbaric treatment for children with autism: a multicenter, randomized, double-blind, controlled trial. *BMC Pediatr* 2009; **9**: 21.

49. Scahill L, McCracken JT, King BH, et al. Extended-Release Guanfacine for Hyperactivity in Children With Autism Spectrum Disorder. *Am J Psychiatry* 2015; **172**(12): 1197-206.

50. Shea S, Turgay A, Carroll A, et al. Risperidone in the treatment of disruptive behavioral symptoms in children with autistic and other pervasive developmental disorders. *Pediatrics* 2004; **114**(5): e634-41.

51. Singh K, Connors SL, Macklin EA, et al. Sulforaphane treatment of autism spectrum disorder (ASD). *Proc Natl Acad Sci U S A* 2014; **111**(43): 15550-5.

52. Sofronoff K, Leslie A, Brown W. Parent management training and Asperger syndrome: a randomized controlled trial to evaluate a parent based intervention. *Autism* 2004; **8**(3): 301-17.

53. Sprengers JJ, van Andel DM, Zuithoff NPA, et al. Bumetanide for Core Symptoms of Autism Spectrum Disorder (BAMBI): A Single Center, Double-Blinded, Participant-Randomized, Placebo-Controlled, Phase-2 Superiority Trial. *J Am Acad Child Adolesc Psychiatry*. 2021; 60(7): 865-876.

54. Veenstra-VanderWeele J, Cook EH, King BH, et al. Arbaclofen in Children and Adolescents with Autism Spectrum Disorder: A Randomized, Controlled, Phase 2 Trial. *Neuropsychopharmacology* 2017; **42**(7): 1390-8.

55. Wasserman S, Iyengar R, Chaplin WF, et al. Levetiracetam versus placebo in childhood and adolescent autism: a double-blind placebo-controlled study. *Int Clin Psychopharmacol* 2006; **21**(6): 363-7.

56. Whittingham K, Sofronoff K, Sheffield J, Sanders MR. Stepping Stones Triple P: an RCT of a parenting program with parents of a child diagnosed with an autism spectrum disorder. *J Abnorm Child Psychol* 2009; **37**(4): 469-80.

57. Wink LK, Adams R, Wang Z, et al. A randomized placebo-controlled pilot study of N-acetylcysteine in youth with autism spectrum disorder. *Mol Autism* 2016; **7**: 26.

58. Wong VC, Chen WX. Randomized controlled trial of electro-acupuncture for autism spectrum disorder. *Altern Med Rev* 2010; **15**(2): 136-46.

59. Yui K, Koshiba M, Nakamura S, Kobayashi Y. Effects of large doses of arachidonic acid added to docosahexaenoic acid on social impairment in individuals with autism spectrum disorders: a double-blind, placebo-controlled, randomized trial. *J Clin Psychopharmacol* 2012; **32**(2): 200-6.

60. Zand DH, Bultas MW, McMillin SE, et al. A Pilot of a Brief Positive Parenting Program on Children Newly Diagnosed with Autism Spectrum Disorder. *Fam Process* 2018; **57**(4): 901-14.

Table S6. The list of excluded articles in the full-text screening and reasons for exclusion (Identification of studies via databases and registers)

Author, year	Title	Exclusion reason
Benton 2011	Aripiprazole to treat irritability associated with autism: a placebo-controlled, fixed-dose trial	Data duplication
Gabriels 2018	Long-Term Effect of Therapeutic Horseback Riding in Youth With Autism Spectrum Disorder: A Randomized Trial	Data duplication
Ichikawa 2018	An open-label extension long-term study of the safety and efficacy of aripiprazole for irritability in children and adolescents with autistic disorder in Japan	Data duplication
Aman 2002	Double-blind, placebo-controlled study of risperidone for the treatment of disruptive behaviors in children with subaverage intelligence	did not enrolled ASD patients for target population
Kirk 2017	Impact of Attention Training on Academic Achievement, Executive Functioning, and Behavior: A Randomized Controlled Trial	did not enrolled ASD patients for target population
Kwak 2020	Findings From a Prospective Randomized Controlled Trial of an Individualized Music Listening Program for Persons With Dementia	did not enrolled ASD patients for target population
McIntyre 2008	Parent training for young children with developmental disabilities: Randomized controlled trial	did not enrolled ASD patients for target population
Ramerman 2019	Is risperidone effective in reducing challenging behaviours in individuals with intellectual disabilities after 1 year or longer use? A placebo-controlled, randomised, double-blind discontinuation study	did not enrolled ASD patients for target population
Adams 2004	Pilot study of a moderate dose multivitamin/mineral supplement for children with autistic spectrum disorder	Did not report irritability scale as an outcome
Allen 2023	Parent-Child Interaction Therapy for Children with Disruptive Behaviors and Autism: A Randomized Clinical Trial	Did not report irritability scale as an outcome
Chugani 2016	Efficacy of Low-Dose Buspirone for Restricted and Repetitive Behavior in Young Children with Autism Spectrum Disorder: A Randomized Trial	Did not report irritability scale as an outcome
de Korte 2021	Pivotal Response Treatment for School-Aged Children and Adolescents with Autism Spectrum Disorder: A Randomized Controlled Trial	Did not report irritability scale as an outcome
Eslamzadeh 2018	Assessment the Efficacy of Atomoxetine in Autism Spectrum Disorders: A Randomized, Double-Blind, Placebo-Controlled Trial	Did not report irritability scale as an outcome
Gordon 1993	A Double-blind Comparison of Clomipramine, Desipramine, and Placebo in the Treatment of Autistic Disorder	Did not report irritability scale as an outcome
Kaale 2012	A randomized controlled trial of preschool-based joint attention intervention for children with autism	Did not report irritability scale as an outcome
Lindgren 2020	A Randomized Controlled Trial of Functional Communication Training via Telehealth for Young Children with Autism Spectrum Disorder	Did not report irritability scale as an outcome
Liu 2019	Effects of Lactobacillus plantarum PS128 on Children with Autism Spectrum Disorder in Taiwan: A Randomized, Double-Blind, Placebo-Controlled Trial	Did not report irritability scale as an outcome
Lopata 2010	RCT of a Manualized Social Treatment for High-Functioning Autism Spectrum Disorders	Did not report irritability scale as an outcome
Mahajan 2022	Efficacy of Risperidone and Methylphenidate for Problem Behaviors and Core Symptoms in Autism Spectrum Disorder: A Randomized Trial	Did not report irritability scale as an outcome
Mankad 2015	A randomized, placebo controlled trial of omega-3 fatty acids in the treatment of young children with autism	Did not report irritability scale as an outcome
Marcus 2011	Safety and tolerability of aripiprazole for irritability in pediatric patients with autistic disorder: a 52-week, open-label, multicenter study	Did not report irritability scale as an outcome
Nekar 2022	Effects of Augmented Reality Game-Based Cognitive-Motor Training on Restricted and Repetitive Behaviors and Executive Function in Patients with Autism Spectrum Disorder	Did not report irritability scale as an outcome
Rohacek 2023	A Preliminary Evaluation of a Brief Behavioral Parent Training for Challenging Behavior in Autism Spectrum Disorder	Did not report irritability scale as an outcome
te Brinke 2022	Treatment Approach and Sequence Effects in Cognitive Behavioral Therapy Targeting Emotion Regulation Among Adolescents with Externalizing Problems and Intellectual Disabilities	Did not report irritability scale as an outcome
Weitlauf 2020	Mindfulness-Based Stress Reduction for Parents Implementing Early Intervention for Autism: An RCT	Did not report irritability scale as an outcome
Wongpakaran 2017	Impact of providing psychiatry specialty pharmacist intervention on reducing drug-related problems among children with autism spectrum disorder related to disruptive behavioural symptoms: A prospective randomized open-label study	Did not report irritability scale as an outcome
Zeng 2021	Effect of the TEACCH program on the rehabilitation of preschool children with autistic spectrum disorder: A randomized controlled trial	Did not report irritability scale as an outcome

Bearss 2015	Effect of Parent Training vs Parent Education on Behavioral Problems in Children With Autism Spectrum Disorder A Randomized Clinical Trial	Inadequate control group
DeVane 2019	Pharmacotherapy of Autism Spectrum Disorder: Results from the Randomized BAART Clinical Trial	Inadequate control group
Iadarola 2018	Teaching Parents Behavioral Strategies for Autism Spectrum Disorder (ASD): Effects on Stress, Strain, and Competence	Inadequate control group
Tellegen 2014	A Randomized Controlled Trial Evaluating a Brief Parenting Program With Children With Autism Spectrum Disorders	Inadequate control group
Carey 2002	Double-Blind Placebo-Controlled Trial of Secretin: Effects on Aberrant Behavior in Children with Autism	Not parallel design
Danfors 2005	Tetrahydrobiopterin in the treatment of children with autistic disorder: A double-blind placebo-controlled crossover study	Not parallel design
Findling 2014	A randomized controlled trial investigating the safety and efficacy of aripiprazole in the long-term maintenance treatment of pediatric patients with irritability associated with autistic disorder	Not parallel design
Marcus 2011	Aripiprazole in the treatment of irritability in pediatric patients (aged 6-17 years) with autistic disorder: results from a 52-week, open-label study	Not parallel design
Posey 2005	Randomized, controlled, crossover trial of methylphenidate in pervasive developmental disorders with hyperactivity	Not parallel design
Research Units on Pediatric Psychopharmacology Autism Network 2005	Risperidone treatment of autistic disorder: longer-term benefits and blinded discontinuation after 6 months	Not parallel design
Scahill 2007	A placebo double-blind pilot study of dextromethorphan for problematic behaviors in children with autism	Not parallel design
Troost 2005	Long-term effects of risperidone in children with autism spectrum disorders: a placebo discontinuation study	Not parallel design
Wink 2018	A Randomized Placebo-Controlled Cross-Over Pilot Study of Riluzole for Drug-Refractory Irritability in Autism Spectrum Disorder	Not parallel design
Campbell 2022	Safety and target engagement of an oral small-molecule sequestrant in adolescents with autism spectrum disorder: an open-label phase 1b/2a trial	Not randomized controlled trial
Cashill 2006	A prospective open trial of guanfacine in children with pervasive developmental disorders	Not randomized controlled trial
Fido 2008	Olanzapine in the treatment of behavioral problems associated with autism: an open-label trial in Kuwait	Not randomized controlled trial
Healy 2018	"I'm not in this alone" the perspective of parents mediating a physical activity intervention for their children with autism spectrum disorder	Not randomized controlled trial
Curran 2011	Aripiprazole: in the treatment of irritability associated with autistic disorder in pediatric patients	Not randomized controlled trial
Erickson 2014	STX209 (Arbaclofen) for Autism Spectrum Disorders: An 8-Week Open-Label Study	Not randomized controlled trial
Gold 2006	Music therapy for autistic spectrum disorder	Not randomized controlled trial
Hurwitz 2012	Tricyclic antidepressants for autism spectrum disorders (ASD) in children and adolescents	Not randomized controlled trial
Jaselskis 1992	Clonidine treatment of hyperactive and impulsive children with autistic disorder	Not randomized controlled trial
Jesner 2007	Risperidone for autism spectrum disorder	Not randomized controlled trial
Jordan 2012	Aripiprazole in the treatment of challenging behaviour in adults with autism spectrum disorder	Not randomized controlled trial
Kolmen 1995	Naltrexone in Young Autistic Children: A Double-Blind, Placebo-Controlled Crossover Study	Not randomized controlled trial
Kuravackel 2018	COMPASS for Hope: Evaluating the Effectiveness of a Parent Training and Support Program for Children with ASD	Not randomized controlled trial
Lewis 2009	Efficacy and Safety of Aripiprazole for the Treatment of Irritability Associated with Autistic Disorder in Children and Adolescents (6-17 Years): Results from Two 8-Week, Randomized, Double-Blind, Placebo-Controlled Trials	Not randomized controlled trial
Lewis 2018	An Exploratory Trial of Transdermal Nicotine for Aggression and Irritability in Adults with Autism Spectrum Disorder	Not randomized controlled trial
Mankoski 2013	Aripiprazole treatment of irritability associated with autistic disorder and the relationship between prior antipsychotic exposure, adverse events, and weight change	Not randomized controlled trial
Miyaoka 2012	Yokukansan (TJ-54) for treatment of pervasive developmental disorder not otherwise specified and Asperger's disorder: A 12-week prospective, open-label study	Not randomized controlled trial

Remington 2001	Clomipramine versus haloperidol in the treatment of autistic disorder: a double-blind, placebo-controlled, crossover study	Not randomized controlled trial
Shiri 2020	A pilot study of family-based management of behavioral excesses in young Iranian children with autism spectrum disorder	Not randomized controlled trial
Solomon 2008	The effectiveness of parent-child interaction therapy for families of children on the autism spectrum	Not randomized controlled trial
Wake 2013	Yokukansan (TJ-54) for irritability associated with pervasive developmental disorder in children and adolescents: a 12-week prospective, open-label study	Not randomized controlled trial
Willemsen-Swinkels 1995	PLACEBO-CONTROLLED ACUTE DOSAGE NALTREXONE STUDY IN YOUNG AUTISTIC-CHILDREN	Not randomized controlled trial
Willemsen-Swinkels 1996	The effects of chronic naltrexone treatment in young autistic children: a double-blind placebo-controlled crossover study	Not randomized controlled trial
Jun 2000	Double blind crossover study of secretin/secrepan treatment for children with autistic symptoms	Not sufficient data
Akhondzadeh 2008	A double-blind placebo controlled trial of piracetam added to risperidone in patients with autistic disorder	Not sufficient data
Anagnostou 2006	Divalproex versus placebo for the prevention of irritability associated with fluoxetine treatment in autism spectrum disorder	Not sufficient data
Belsito 2001	Lamotrigine therapy for autistic disorder: a randomized, double-blind, placebo-controlled trial	Not sufficient data
Campbell 1988	Efficacy and Safety of Fenfluramine in Autistic Children	Not sufficient data
Carminati 2016	Using venlafaxine to treat behavioral disorders in patients with autism spectrum disorder	Not sufficient data
Hassiotis 2018	Clinical outcomes of staff training in positive behaviour support to reduce challenging behaviour in adults with intellectual disability: Cluster randomised controlled trial	Not sufficient data
Hollander 2006	Divalproex sodium vs. placebo in the treatment of repetitive behaviours in autism spectrum disorder	Not sufficient data
Johnson 2010	Polyunsaturated fatty acid supplementation in young children with autism	Not sufficient data
Jung 2000	A double blind study of dimethylglycine treatment in children with autism	Not sufficient data
Klaiman 2013	Tetrahydrobiopterin as a treatment for autism spectrum disorders: A double-blind, placebo-controlled trial	Not sufficient data
Liang 2020	Effectiveness of parent-training program on children with autism spectrum disorder in China	Not sufficient data
Luby 2006	Risperidone in preschool children with autistic spectrum disorders: an investigation of safety and efficacy	Not sufficient data
McDougle 1996	A double-blind, placebo-controlled study of fluvoxamine in adults with autistic disorder	Not sufficient data
Nagaraj 2006	Risperidone in children with autism: randomized, placebo-controlled, double-blind study	Not sufficient data
Scudder 2019	Parent-child interaction therapy (PCIT) in young children with autism spectrum disorder	Not sufficient data
Shooshtari 2020	The Effect of a Parental Education Program on the Mental Health of Parents and Behavioral Problems of Their Children With Autism Spectrum Disorder	Not sufficient data
Strain 2011	Randomized, Controlled Trial of the LEAP Model of Early Intervention for Young Children With Autism Spectrum Disorders	Not sufficient data
Willemsen-Swinkels 1995	Failure of naltrexone hydrochloride to reduce self-injurious and autistic behavior in mentally retarded adults. Double-blind placebo-controlled studies	Not sufficient data
Zimmerman 2021	Randomized controlled trial of sulforaphane and metabolite discovery in children with Autism Spectrum Disorder	Not sufficient data

Table S7. The list of excluded articles in the full-text screening and reasons for exclusion (Identification of studies via other methods)

Author, year	Title	Exclusion reason
Amminger 2007	Omega-3 fatty acids supplementation in children with autism: a double-blind randomized, placebo-controlled pilot study	Already found
Belsito 2001	Lamotrigine therapy for autistic disorder: a randomized, double-blind, placebo-controlled trial	Already found
Bent 2011	A pilot randomized controlled trial of omega-3 fatty acids for autism spectrum disorder	Already found
Bent 2014	Internet-based, randomized, controlled trial of omega-3 fatty acids for hyperactivity in autism	Already found
Findling 1997	High-dose pyridoxine and magnesium administration in children with autistic disorder: an absence of salutary effects in a double-blind, placebo-controlled study	Already found
Ginn 2017	Child-Directed Interaction Training for Young Children With Autism Spectrum Disorders: Parent and Child Outcomes	Already found
Handen 2015	Atomoxetine, Parent Training, and Their Combination in Children With Autism Spectrum Disorder and Attention-Deficit/Hyperactivity Disorder	Already found
Klaiman 2013	Tetrahydrobiopterin as a treatment for autism spectrum disorders: a double-blind, placebo-controlled trial	Already found
Kuravackel 2018	COMPASS for Hope: Evaluating the Effectiveness of a Parent Training and Support Program for Children with ASD	Already found
Singh 2014	Sulforaphane treatment of autism spectrum disorder (ASD)	Already found
Sofronoff 2004	Parent management training and Asperger syndrome: a randomized controlled trial to evaluate a parent based intervention	Already found
Solomon 2008	The effectiveness of parent-child interaction therapy for families of children on the autism spectrum	Already found
Tellegen 2014	A randomized controlled trial evaluating a brief parenting program with children with autism spectrum disorders	Already found
Whittingham 2009	Stepping Stones Triple P: an RCT of a parenting program with parents of a child diagnosed with an autism spectrum disorder	Already found
Yui 2012	Effects of large doses of arachidonic acid added to docosahexaenoic acid on social impairment in individuals with autism spectrum disorders: a double-blind, placebo-controlled, randomized trial	Already found
Zand 2018	A Pilot of a Brief Positive Parenting Program on Children Newly Diagnosed with Autism Spectrum Disorder	Already found
Bearss 2015	Effect of parent training vs parent education on behavioral problems in children with autism spectrum disorder: a randomized clinical trial	Already found
Bolman 1999	A double-blind, placebo-controlled, crossover pilot trial of low dose dimethylglycine in patients with autistic disorder	Not sufficient data
Kern 2001	Effectiveness of N,N-dimethylglycine in autism and pervasive developmental disorder	Not sufficient data
Adams 2011	Effect of a vitamin/mineral supplement on children and adults with autism	Did not report irritability scale as an outcome

Table S8. Meta-regression analyses for (1) publication year, (2) sample size, (3) mean age of intervention group, and (4) male percentage of the intervention group.

[Note]

- Meta-regression analysis was available only for meta-analysis with more than three studies.
- We highlighted a statistically significant moderator with a star.

1. Pharmacological monotherapy

Intervention	Potential moderators	k	Coefficient (95% CI)	*P*	NA reason
**Risperidone**					
	Publication year	6	0.0494 (-0.0169 to 0.1158)	0.1074	
	Sample size	6	-0.0066 (-0.0286 to 0.0154)	0.4535	
	Mean age of intervention group	4	0.1117 (-0.3675 to 0.5910)	0.4215	
	Male percentage of intervention group	5	-0.0023 (-0.0925 to 0.0878)	0.9397	
**Aripiprazole**					
	Publication year	5	0.0211 (-0.0579 to 0.1001)	0.4575	
	Sample size	5	0.0040 (-0.0444 to 0.0524)	0.8096	
	Mean age of intervention group	5	0.0111 (-0.6085 to 0.6308)	0.9580	
	Male percentage of intervention group	5	-0.0243 (-0.0911 to 0.0425)	0.3313	
**Lurasidone**					k<4
**Anti-epileptic drug**					k<4
**Valproate**					k<4

Abbreviations: CI, confidence interval; k, the number of studies; NA, not available.

2. Risperidone plus adjuvant therapy vs. risperidone

Intervention	Potential moderators	k	Coefficient (95% CI)	*P*	NA reason
**Risperidone + dietary supplementation vs. risperidone**					
	Publication year	5	-0.0655 (-0.2707 to 0.1377)	0.3763	
	Sample size	5	-0.0138 (-0.0861 to 0.0585)	0.5871	
	Mean age of intervention group	5	0.0025 (-0.7541 to 0.7590)	0.9924	
	Male percentage of intervention group	5	0.0177 (-0.0627 to 0.0981)	0.5338	
**Risperidone + N-acetylcysteine vs risperidone**					k<4

Abbreviations: CI, confidence interval; k, the number of studies; NA, not available.

3. Non-pharmacological intervention

Intervention	Potential moderators	k	Coefficient (95% CI)	*P*	NA reason
**Parent training**					
	Publication year	6	0.0137 (-0.0499 to 0.0774)	0.5812	
	Sample size	6	0.0056 (-0.0209 to 0.0321)	0.5902	
	Mean age of intervention group	4	0.1793 (-0.0656 to 0.4243)	0.0877	
	Male percentage of intervention group	4	0.0057 (-0.2118 to 0.2233)	0.9202	
**Stepping Stone Triple P**					k<4

Abbreviations: CI, confidence interval; k, the number of studies; NA, not available.

4. Dietary intervention

Intervention	Potential moderators	k	Coefficient (95% CI)	*P*	NA reason
**Polyunsaturated fatty acid**					
	Publication year	5	-0.1048 (-0.2393 to 0.0298)	0.0894	
	Sample size	5	-0.0039 (-0.0501 to 0.0423)	0.8070	
	Mean age of intervention group	4	0.1690 (-0.4683 to 0.8062)	0.3722	
	Male percentage of intervention group				k<4
**Omega-3 fatty acid**					
	Publication year	4	-0.1019 (-0.3219 to 0.1181)	0.1845	
	Sample size	4	-0.0028 (-0.0890 to 0.0834)	0.9012	
	Mean age of intervention group				k<4
	Male percentage of intervention group				k<4
**N-acetylcysteine**					k<4
**Vitamin D3**					k<4

Abbreviations: CI, confidence interval; k, the number of studies; NA, not available.

Table S9. Subgroup analyses for (1) RoB2 and (2) measurement tool for irritability

[Note]

- We highlighted a statistically significant moderator with a star.

1. Pharmacological monotherapy

Intervention	Potential moderators		k	Hedges'g (95% CI)	Q	I^2	*P*	NA reason
**Risperidone**								
	RoB2						0.5968	
		Low	3	-0.8775 (-2.1987 to 0.4436)	9.95	79.9		
		Some concerns	2	-0.7101 (-1.5471 to 0.1269)	0.13	0.0		
		High	1	-1.1910 (-2.2568 to -0.1252)	-	-		
	Measurement tool for irritability						0.4840	
		ABC-I	5	-0.8148 (-1.3044 to -0.3253)	10.77	62.9		
		SIB-Q	1	-1.1910 (-2.1861 to -0.1959)	-	-		
**Aripiprazole**		NA						All included studies were low risk of bias and used the same measurement tool for irritability.
**Lurasidone**		NA						All included studies were low risk of bias and used the same measurement tool for irritability.
**Anti-epileptic drug**								
	RoB2						0.3083	
		Low	2	-0.3762 (-4.0836 to 3.3311)	0.96	0.0		
		Some concerns	1	0.1290 (-0.6569 to 0.9149)	-	-		
	Measurement tool for irritability	NA						All included studies used the same measurement tool for irritability
**Valproate**								
	RoB2						0.1762	
		Low	1	-0.6380 (-1.4239 to 0.1479)	-	-		
		Some concerns	1	0.1290 (-0.6569 to 0.9149)	-	-		
	Measurement tool for irritability	NA						All included studies used the same measurement tool for irritability

Abbreviations: ABC-I, the Aberrant Behavior Checklist-Irritability; CI, confidence interval; k, the number of studies; NA, not available; RoB2, risk of bias 2; SIB-Q, Self-Injurious Behavior Questionnaire

2. Risperidone plus adjuvant therapy vs. risperidone

Intervention	Potential moderators		k	Hedges'g (95% CI)	Q	I^2	*P*	NA reason
**Risperidone + dietary supplementation vs. risperidone**								
	RoB2						0.7028	
		Low	3	-0.4153 (-1.5351 to 0.7044)	4.84	58.6		
		Some concerns	2	-0.6038 (-5.9373 to 4.7298)	3.38	70.4		
	Measurement tool for irritability	NA						All included studies used the same measurement tool for irritability
**Risperidone + N-acetylcysteine vs. risperidone only**								
	RoB2						0.1320	
		Low	1	-0.2920 (-1.0035 to 0.4195)	-	-		
		Some concerns	1	-1.0380 (-1.6985 to -0.3775)	-	-		
	Measurement tool for irritability	NA						All included studies used the same measurement tool for irritability

Abbreviations: CI, confidence interval; k, the number of studies; NA, not available; RoB2, risk of bias 2.

3. Non-pharmacological intervention

Intervention	Potential moderators		k	Hedges'g (95% CI)	Q	I^2	*P*	NA reason
**Parent training**								
	RoB2						0.1022	
		High	5	-1.0057 (-1.2255 to -0.7859)	0.97	0.0		
		Some concerns	1	-0.4970 (-1.0869 to 0.0929)	-	-		
	Measurement tool for irritability						0.1022	
		ECBI intensity	5	-1.0057 (-1.2255 to -0.7859)	0.97	0.0		
		ABC-I	1	-0.4970 (-1.0869 to 0.0929)	-	-		
**Stepping Stone Triple P**		NA						All included studies were high risk of bias and used the same measurement tool for irritability

Abbreviations: ABC-I, the Aberrant Behavior Checklist-Irritability; CI, confidence interval; ECBI, The Eyberg Child Behavior Inventory; k, the number of studies; NA, not available; RoB2, risk of bias 2.

4. Dietary intervention

Intervention	Potential moderators		k	Hedges'g (95% CI)	Q	I^2	*P*	NA reason
**N-acetylcysteine**								
	RoB2						0.3438	
		Low	2	-0.3927 (-6.1085 to 5.3231)	4.27	76.6		
		Some concerns	1	0.3700 (-0.9401 to 1.6801)	-	-		
	Measurement tool for irritability						0.8057	
		ABC-I	2	-0.2608 (-8.3794 to 7.8578)	5.77	82.7		
		DBC-Irritable	1	0.0000 (-1.6583 to 1.6583)				
**Polyunsaturated fatty acid**								
	RoB2						**0.0026***	
		Low	4	0.0169 (-0.0389 to 0.0727)	0.02	0.0		
		Some concerns	1	-1.0280 (-1.7061 to -0.3499)	-	-		
	Measurement tool for irritability	NA						All included studies used the same measurement tool for irritability
**Omega-3 fatty acid**								
	RoB2						**0.0024***	
		Low	3	0.0246 (-0.0473 to 0.0965)	0.01	0.0		
		Some concerns	1	-1.0280 (-1.7061 to -0.3499)	-	-		
	Measurement tool for irritability	NA						All included studies used the same measurement tool for irritability
**Vitamin D3**								
	RoB2						**0.0224***	
		Low	1	0.2430 (-0.3959 to 0.8819)	-	-		
		Some concerns	1	-0.8560 (-1.5498 to -0.1622)	-	-		
	Measurement tool for irritability	NA						All included studies used the same measurement tool for irritability

Abbreviations: ABC-I, the Aberrant Behavior Checklist-Irritability; CI, confidence interval; DBC, Developmental Behavior Checklist; k, the number of studies; NA, not available; RoB2, risk of bias 2.

Figure S1. Forest plot and funnel plot for each meta-analysis

I. Pharmacological intervention

1. Risperidone

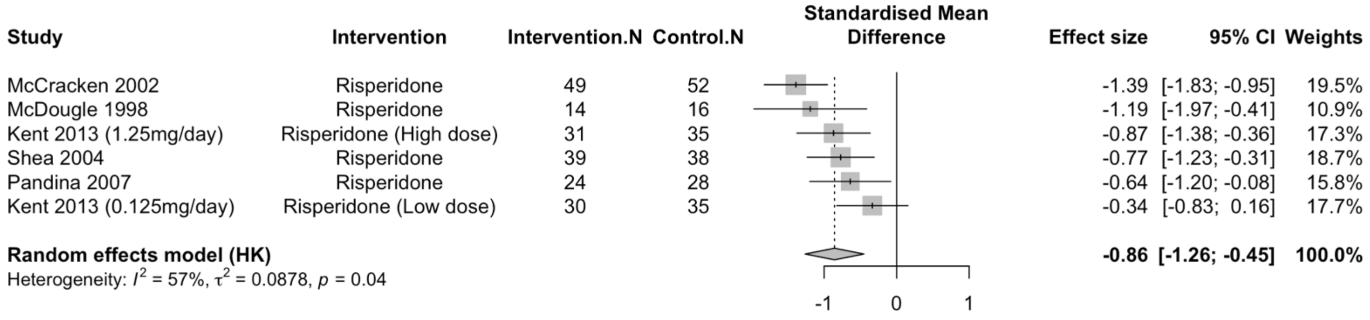

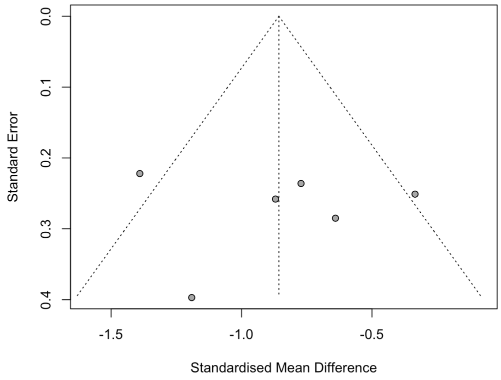

2. Aripiprazole

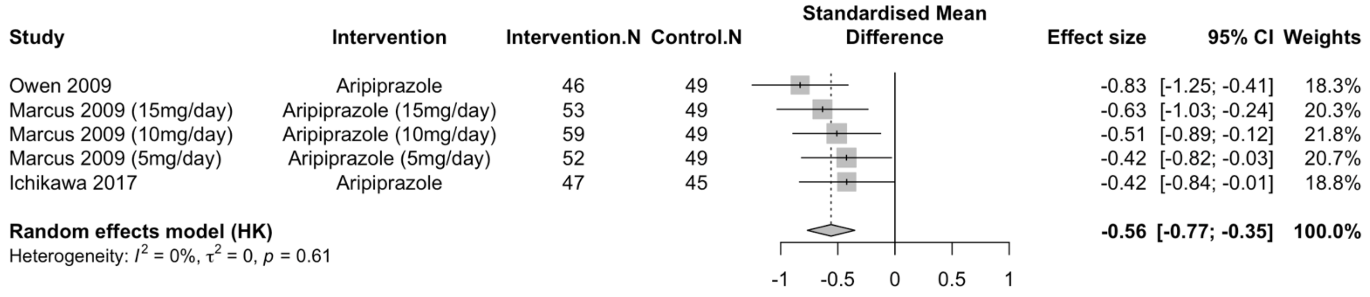

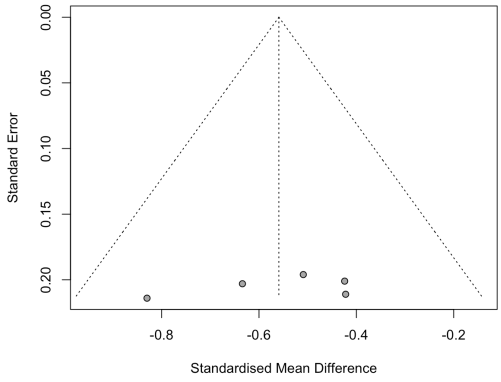

3. Lurasidone

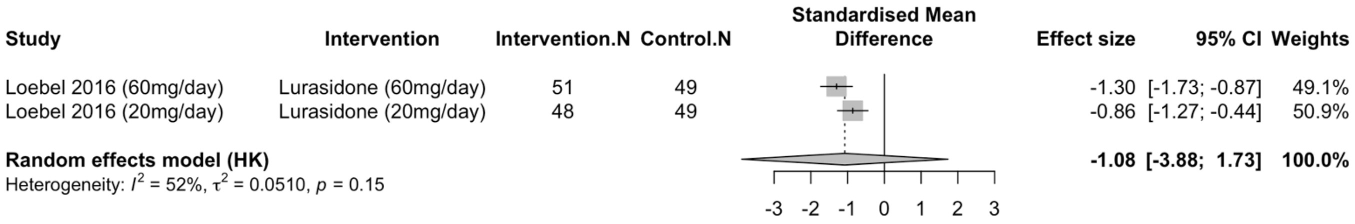

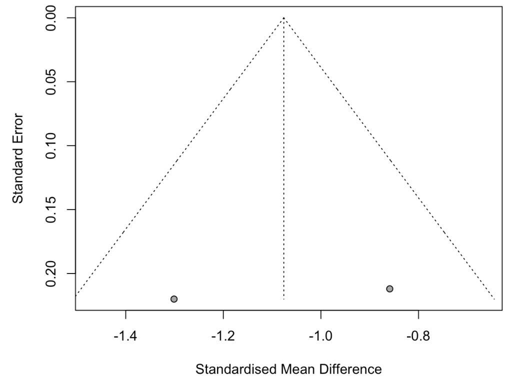

4. Anti-epileptic drugs

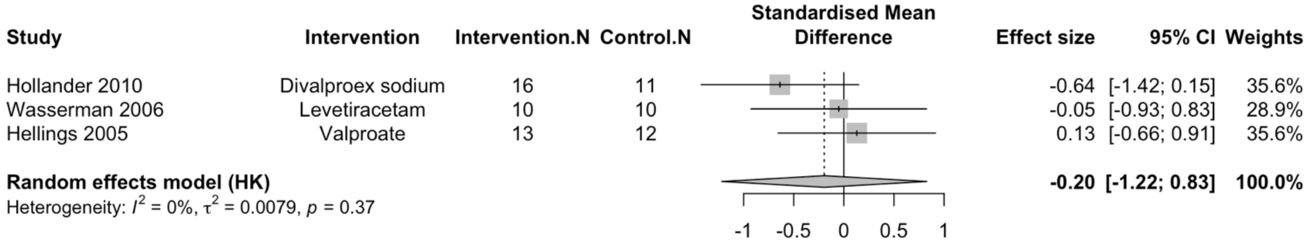

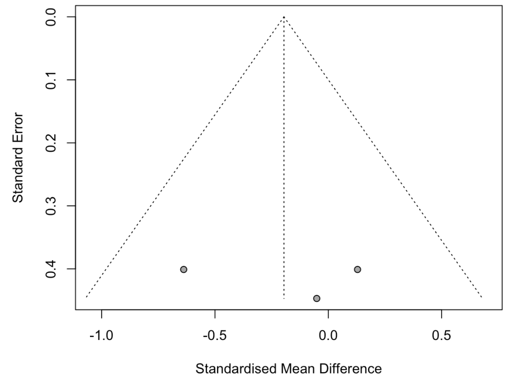

5. Valproate

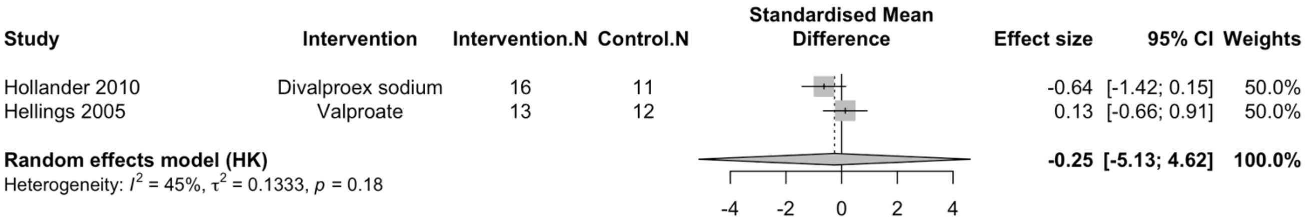
Study
Intervention
Intervention.N
Control.N
Standardised Mean Difference
Effect size
95% CI
Weights
Hollander 2010
Divalproex sodium
16
11
-0.64
[-1.42; 0.15]
50.0%
Hellings 2005
Valproate
13
12
0.13
[-0.66; 0.91]
50.0%
Random effects model (HK)
-0.25
[-5.13; 4.62]
100.0%
Heterogeneity: I^2 = 45%, τ^2 = 0.1333, p = 0.18
-4
-2
0
2
4

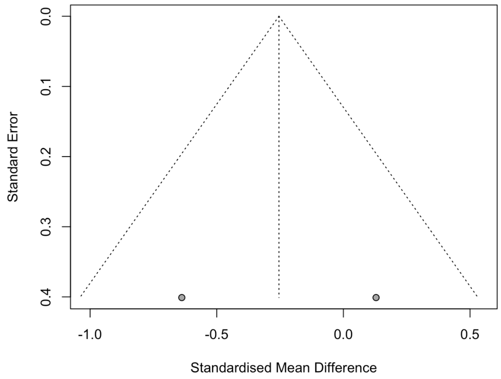
Standard Error
0.0
0.1
0.2
0.3
0.4
-1.0
-0.5
0.0
0.5
Standardised Mean Difference

II. Risperidone plus adjuvant therapy vs. risperidone

1. Risperidone + dietary supplementation vs. risperidone

Study	Intervention	Intervention.N	Control.N	Effect size	95% CI	Weights
Nikoo 2015	Risperidone+N-Acetylcysteine vs control	20	20	-1.04	[-1.70; -0.38]	18.6%
Momtazmanesh 2020	Risperidone+Sulforaphane vs control	30	30	-0.88	[-1.41; -0.35]	22.8%
Ghanizadeh 2013	Risperidone+N-Acetylcysteine vs control	17	14	-0.29	[-1.00; 0.42]	17.1%
Hajizadeh-Zaker 2018	Risperidone+L-Carnosine vs control	21	21	-0.20	[-0.80; 0.41]	20.2%
Hasanzadeh 2012	Risperidone+Ginkgo biloba vs control	23	24	-0.03	[-0.60; 0.54]	21.3%
**Random effects model (HK)**				**-0.49**	**[-1.05; 0.07]**	**100.0%**

Heterogeneity: $I^2 = 53\%$, $\tau^2 = 0.1067$, $p = 0.08$

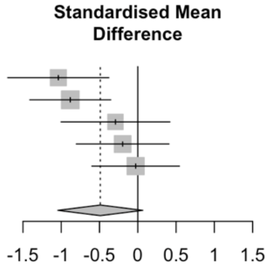

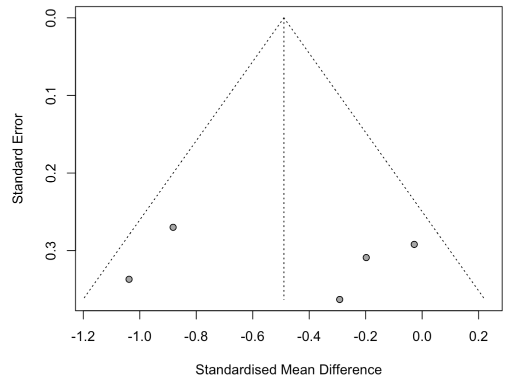

2. Risperidone + N-acetylcysteine vs. risperidone

Study	Intervention	Intervention.N	Control.N	Effect size	95% CI	Weights
Nikoo 2015	Risperidone+N-Acetylcysteine vs control	20	20	-1.04	[-1.70; -0.38]	51.6%
Ghanizadeh 2013	Risperidone+N-Acetylcysteine vs control	17	14	-0.29	[-1.00; 0.42]	48.4%
**Random effects model (HK)**				**-0.68**	**[-5.41; 4.06]**	**100.0%**

Heterogeneity: $I^2 = 56\%$, $\tau^2 = 0.1556$, $p = 0.13$

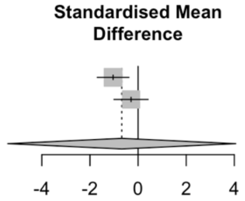

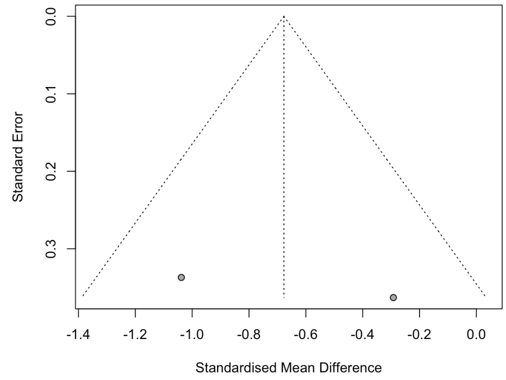

III. Non-pharmacological intervention

1. Parent training

Study	Intervention	Intervention.N	Control.N	Effect size	95% CI	Weights
Sofronoff 2004	Parent Management Training-Individual	18	15	-1.27	[-2.02; -0.52]	13.7%
Ginn 2017	Child-Directed Interaction Training	15	15	-1.07	[-1.83; -0.30]	13.2%
Zand 2018	Stepping Stones Triple P	12	9	-1.04	[-1.97; -0.12]	9.1%
Whittingham 2009	Stepping Stones Triple P	29	30	-0.96	[-1.50; -0.42]	26.5%
Sofronoff 2004	Parent Management Training-Workshop	18	15	-0.77	[-1.48; -0.06]	15.3%
Handen 2015	Parent training	25	21	-0.50	[-1.09; 0.09]	22.2%
**Random effects model (HK)**				**-0.89**	**[-1.18; -0.60]**	**100.0%**

Heterogeneity: $I^2 = 0\%$, $\tau^2 = 0$, $p = 0.67$

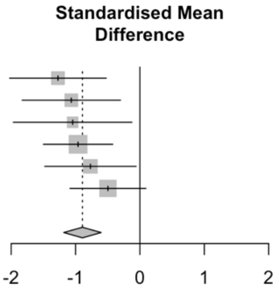

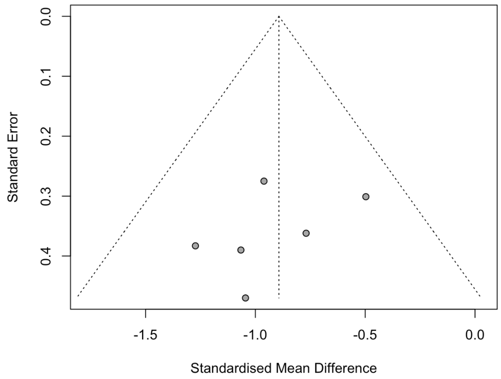

2. Stepping Stones Triple P

Study	Intervention	Intervention.N	Control.N	Effect size	95% CI	Weights
Zand 2018	Stepping Stones Triple P	12	9	-1.04	[-1.97; -0.12]	25.5%
Whittingham 2009	Stepping Stones Triple P	29	30	-0.96	[-1.50; -0.42]	74.5%
**Random effects model (HK)**				**-0.98**	**[-1.45; -0.52]**	**100.0%**

Heterogeneity: $I^2 = 0\%$, $\tau^2 = 0$, $p = 0.88$

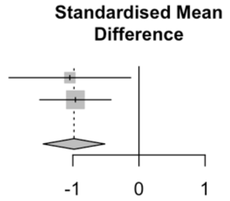

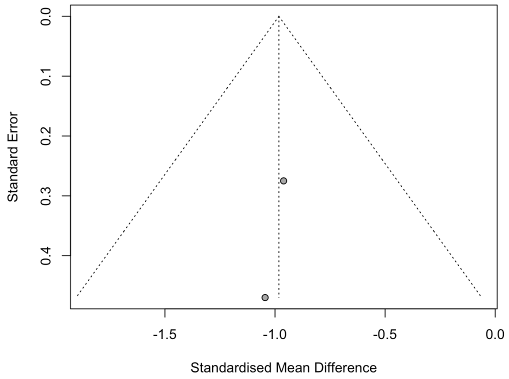

IV. Dietary intervention

1. N-acetylcysteine

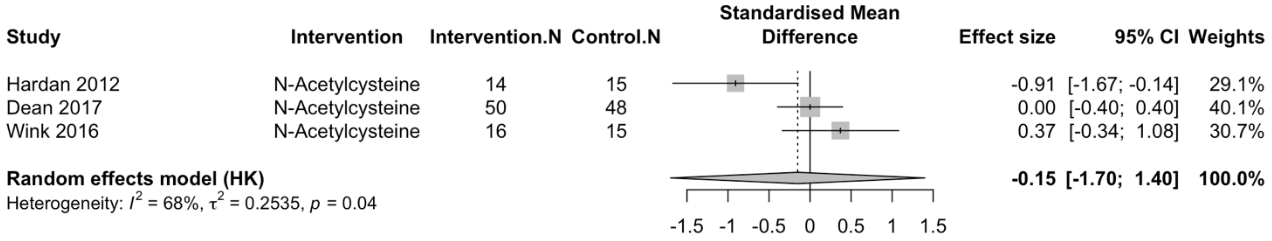

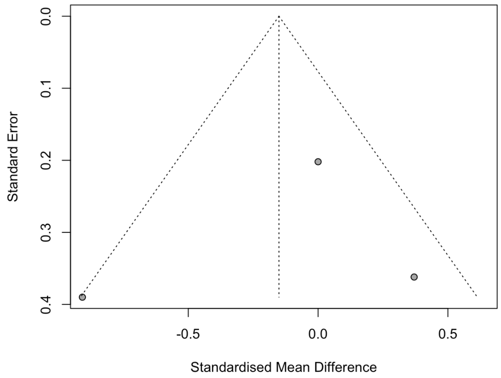

2. Polyunsaturated fatty acid

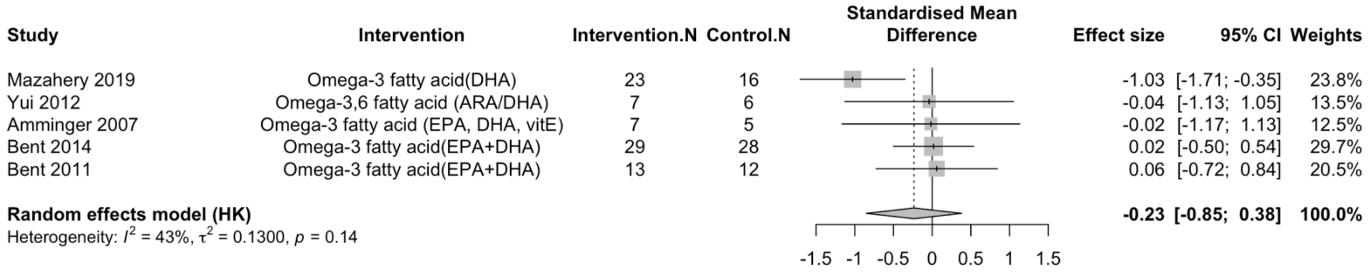

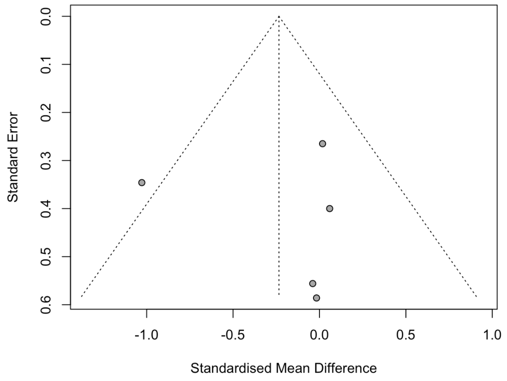

3. Omega-3 fatty acid

Study	Intervention	Intervention.N	Control.N	Effect size	95% CI	Weights
Mazahery 2019	Omega-3 fatty acid(DHA)	23	16	-1.03	[-1.71; -0.35]	27.4%
Amminger 2007	Omega-3 fatty acid (EPA, DHA, vitE)	7	5	-0.02	[-1.17; 1.13]	15.6%
Bent 2014	Omega-3 fatty acid(EPA+DHA)	29	28	0.02	[-0.50; 0.54]	32.8%
Bent 2011	Omega-3 fatty acid(EPA+DHA)	13	12	0.06	[-0.72; 0.84]	24.1%
**Random effects model (HK)**				**-0.26**	**[-1.13; 0.60]**	**100.0%**

Heterogeneity: $I^2 = 56\%$, $\tau^2 = 0.1782$, $p = 0.08$

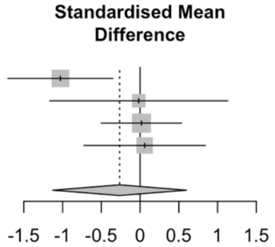

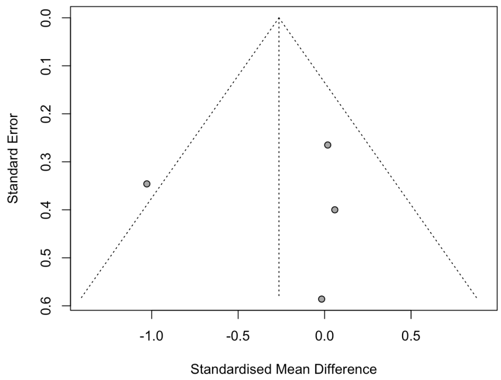

4. Vitamin D_3

Study	Intervention	Intervention.N	Control.N	Effect size	95% CI	Weights
Mazahery 2019	Vitamin D3	19	16	-0.86	[-1.55; -0.16]	49.2%
Kerley 2017	Vitamin D3	18	20	0.24	[-0.40; 0.88]	50.8%
**Random effects model (HK)**				**-0.30**	**[-7.28; 6.68]**	**100.0%**

Heterogeneity: $I^2 = 81\%$, $\tau^2 = 0.4881$, $p = 0.02$

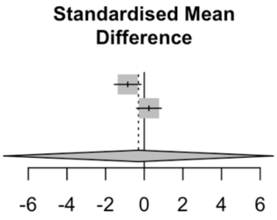

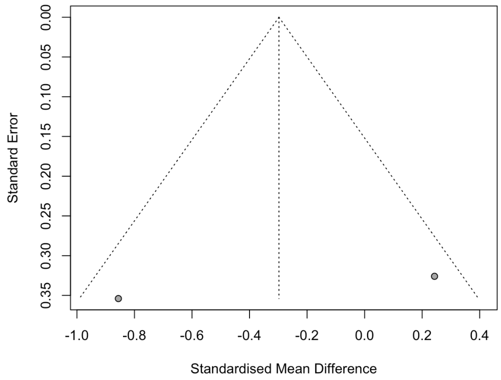

Table S10. Details of risk of bias 2 assessment

Akhondzadeh 2010, Double-blind placebo-controlled trial of pentoxifylline added to risperidone: effects on aberrant behavior in children with autism

Bias	Author’s judgement	Support for judgement
Bias arising from the randomization process	Low risk	Allocation sequence seems to be concealed to participants. Baseline characteristics suggests no problem.
Bias due to deviations from intended interventions	Low risk	Participants were unaware of the intervention. Appropriate analysis was taken, thus no deviations from the intended interventions is expected.
Bias due to missing outcome data	Low risk	There was no missing data.
Bias in measurement of the outcome	Low risk	Outcome measurement was consistent in both intervention groups, and investigators were unaware of the intervention patients received.
Bias in selection of the reported result	Low risk	Reported results for a single outcome measurement which is clearly defined correspond to intended analyses, and it seems to be based on the protocol.

Amminger 2007, Omega-3 fatty acids supplementation in children with autism: A double-blind randomized, placebo-controlled pilot study

**Bias**	**Author's judgement**	**Support for judgement**
Bias arising from the randomization process	Low risk	Allocation sequence seems to be concealed to participants, and no detailed information was presented on randomization method. Baseline characteristics suggests no problem.
Bias due to deviations from intended interventions	Low risk	Participants were unaware of the drug assignment. Appropriate analysis was taken, thus no deviations from the intended interventions is expected.
Bias due to missing outcome data	Low risk	Outcome data were available for nearly all randomized participants.
Bias in measurement of the outcome	Low risk	Outcome measurement was consistent in both intervention groups, and investigators were unaware of the intervention patients received.
Bias in selection of the reported result	Low risk4	Reported results for multiple outcome measurements which is clearly defined correspond to intended analyses, and it seems to be based on the protocol.

Arnold 2012, Placebo-controlled pilot trial of mecamylamine for treatment of autism spectrum disorders

**Bias**	**Author's judgement**	**Support for judgement**
Bias arising from the randomization process	Some concerns	Allocation sequence seems to be concealed to participants. Baseline characteristics suggests some problem.
Bias due to deviations from intended interventions	Low risk	Participants were unaware of the intervention. Appropriate analysis (ITT analysis) was taken, thus no deviations from the intended interventions is expected.
Bias due to missing outcome data	Low risk	10% (2 out of 20) participants were missed, and they took 'last-observation-carried-forward' method which could not correct the bias. Though, the missingness do not depend on true value.
Bias in measurement of the outcome	Low risk	Outcome measurement was consistent in both intervention groups, and investigators were unaware of the intervention patients received.
Bias in selection of the reported result	Low risk	Reported results for multiple outcome measurements which is clearly defined correspond to intended analyses, and it seems to be based on the protocol.

Asadabadi 2013, Celecoxib as adjunctive treatment to risperidone in children with autistic disorder: a randomized, double-blind, placebo-controlled trial

Bias	Author's judgement	Support for judgement
Bias arising from the randomization process	Low risk	Allocation sequence was adequately concealed. Baseline characteristics suggests no problem.
Bias due to deviations from intended interventions	Low risk	Participants were unaware of the intervention. Appropriate analysis was taken, thus no deviations from the intended interventions is expected.
Bias due to missing outcome data	Low risk	There was no missing data.
Bias in measurement of the outcome	Low risk	Outcome measurement was consistent in both intervention groups, and investigators were unaware of the intervention patients received.
Bias in selection of the reported result	Low risk	Reported results for a single outcome measurement which is clearly defined correspond to intended analyses, and it seems to be based on the protocol.

Ayatollahi 2020, Does pregnenolone adjunct to risperidone ameliorate irritable behavior in adolescents with autism spectrum disorder: A randomized, double-blind, placebo-controlled clinical trial?

**Bias**	**Author's judgement**	**Support for judgement**
Bias arising from the randomization process	Low risk	Allocation sequence seems to be concealed to participants. Baseline characteristics suggests no problem.
Bias due to deviations from intended interventions	Low risk	Participants were unaware of the intervention. Appropriate analysis was taken, thus no deviations from the intended interventions is expected.
Bias due to missing outcome data	Low risk	7.8% (5 out of 64) participants were missed, and there was no information about the effort to account the bias. Though, missingness do not seem to depend on true value.
Bias in measurement of the outcome	Low risk	Outcome measurement was consistent in both intervention groups, and investigators were unaware of the intervention patients received.
Bias in selection of the reported result	Low risk	Reported results for multiple outcome measurements which is clearly defined correspond to intended analyses, and it seems to be based on the protocol.

Behmanesh 2019, Risperidone Combination Therapy With Propentofylline for Treatment of Irritability in Autism Spectrum Disorders: A Randomized, Double-Blind, Placebo-Controlled Clinical Trial

Bias	Author's judgement	Support for judgement
Bias arising from the randomization process	Low risk	Allocation sequence adequately concealed to participants. Baseline characteristics suggests no problem.
Bias due to deviations from intended interventions	Low risk	Participants were unaware of the intervention. Appropriate analysis was taken, thus no deviations from the intended interventions is expected.
Bias due to missing outcome data	Low risk	11% (7 out of 62) participants were missed, and there was no information about the effort to account the bias. Though, missingness do not seem to depend on true value.
Bias in measurement of the outcome	Low risk	Outcome measurement was consistent in both intervention groups, and investigators were unaware of the intervention patients received.
Bias in selection of the reported result	Low risk	Reported results for multiple outcome measurements which is clearly defined correspond to intended analyses, and it seems to be based on the protocol.

Bent 2011, A pilot randomized controlled trial of omega-3 fatty acids for autism spectrum disorder

**Bias**	**Author's judgement**	**Support for judgement**
Bias arising from the randomization process	Low risk	Allocation sequence was adequately concealed. Baseline characteristics suggests no problem.
Bias due to deviations from intended interventions	Low risk	Participants were unaware of the intervention. Appropriate analysis (ITT analysis) was taken, thus no deviations from the intended interventions is expected.
Bias due to missing outcome data	Low risk	7.4% (2 out of 27) participants were missed, but there was an effort to account the bias.
Bias in measurement of the outcome	Low risk	Outcome measurement was consistent in both intervention groups, and investigators were unaware of the intervention patients received.
Bias in selection of the reported result	Low risk	Reported results for multiple outcome measurements which is clearly defined correspond to intended analyses, and it seems to be based on the protocol.

Bent 2014, Internet-based, randomized, controlled trial of omega-3 fatty acids for hyperactivity in autism

Bias	Author's judgement	Support for judgement
Bias arising from the randomization process	Low risk	Allocation sequence was adequately concealed. Baseline characteristics suggests no problem.
Bias due to deviations from intended interventions	Low risk	Participants were unaware of the intervention. Appropriate analysis (ITT analysis) was taken, thus no deviations from the intended interventions is expected.
Bias due to missing outcome data	Low risk	There was no missing data.
Bias in measurement of the outcome	Low risk	Outcome measurement was consistent in both intervention groups, and investigators were unaware of the intervention patients received.
Bias in selection of the reported result	Low risk	Reported results for multiple outcome measurements which is clearly defined correspond to intended analyses, and it seems to be based on the protocol.

Dean 2017, A randomised, double blind, placebo-controlled trial of a fixed dose of N -acetyl cysteine in children with autistic disorder

Bias	Author's judgement	Support for judgement
Bias arising from the randomization process	Low risk	Allocation sequence was adequately concealed. Baseline characteristics suggests no problem.
Bias due to deviations from intended interventions	Low risk	Participants were unaware of the intervention. Appropriate analysis (ITT analysis) was taken, thus no deviations from the intended interventions is expected.
Bias due to missing outcome data	Low risk	30% (31 out of 102) participants were missed, and there was no information about the effort to account the bias. Though, missingness do not seem to depend on true value.
Bias in measurement of the outcome	Low risk	Outcome measurement was consistent in both intervention groups, and investigators were unaware of the intervention patients received.
Bias in selection of the reported result	Low risk	Reported results for multiple outcome measurements which is clearly defined correspond to intended analyses, and it seems to be based on the protocol.

Frye 2016, Folinic acid improves verbal communication in children with autism and language impairment: a randomized double-blind placebo-controlled trial

**Bias**	**Author's judgement**	**Support for judgement**
Bias arising from the randomization process	Low risk	Allocation sequence seems to be concealed to participants. Baseline characteristics suggests no problem.
Bias due to deviations from intended interventions	Low risk	Participants were unaware of the intervention. Appropriate analysis (ITT analysis) was taken, thus no deviations from the intended interventions is expected.
Bias due to missing outcome data	Low risk	10.4% (5 out of 48) participants were missed. Though, evidence that the result was not biased by missing data comes from sensitivity analysis.
Bias in measurement of the outcome	Low risk	Outcome measurement was consistent in intervention groups, and raters were unaware of the intervention patients received.
Bias in selection of the reported result	Low risk	Reported results for multiple outcome measurements which is clearly defined correspond to intended analyses, and it seems to be based on the protocol.

Gabriels 2015, Randomized Controlled Trial of Therapeutic Horseback Riding in Children and Adolescents With Autism Spectrum Disorder

Bias	Author's judgement	Support for judgement
Bias arising from the randomization process	Low risk	Allocation sequence was adequately concealed. Baseline characteristics suggests no problem.
Bias due to deviations from intended interventions	High risk	Participants were aware of the intervention, and an appropriate analysis was not used to estimate the effect of adhering to the intervention
Bias due to missing outcome data	Low risk	24% (30 out of 127) participants were missed, but there was an effort to account the bias.
Bias in measurement of the outcome	Some concerns	Outcome measurement was consistent in both intervention groups, but investigators were aware of the intervention patients received which could have influenced by knowledge of intervention.
Bias in selection of the reported result	Low risk	Reported results for multiple outcome measurements which is clearly defined correspond to intended analyses, and it seems to be based on the protocol.

Ghaleiha 2013a, Memantine as adjunctive treatment to risperidone in children with autistic disorder: a randomized, double-blind, placebo-controlled trial

**Bias**	**Author's judgement**	**Support for judgement**
Bias arising from the randomization process	Low risk	Allocation sequence seems to be concealed to participants. Baseline characteristics suggests no problem.
Bias due to deviations from intended interventions	Low risk	Participants were unaware of the intervention. Appropriate analysis was taken, thus no deviations from the intended interventions is expected.
Bias due to missing outcome data	Low risk	There was no missing data.
Bias in measurement of the outcome	Low risk	Outcome measurement was consistent in both intervention groups, and investigators were unaware of the intervention patients received.
Bias in selection of the reported result	Low risk	Reported results for a single outcome measurement which is clearly defined correspond to intended analyses, and it seems to be based on the protocol.

Ghaleiha 2013b, Riluzole as an Adjunctive Therapy to Risperidone for the Treatment of Irritability in Children with Autistic Disorder: A Double-Blind, Placebo-Controlled, Randomized Trial

**Bias**	**Author's judgement**	**Support for judgement**
Bias arising from the randomization process	Low risk	Allocation sequence was adequately concealed. Baseline characteristics suggests no problem.
Bias due to deviations from intended interventions	Low risk	Participants were unaware of the intervention. Appropriate analysis (ITT analysis) was taken, thus no deviations from the intended interventions is expected.
Bias due to missing outcome data	Some concerns	18% (9 out of 49) participants were missed, and there was no information about the effort to account the bias. Some missingness could depend on true value.
Bias in measurement of the outcome	Low risk	Outcome measurement was consistent in both intervention groups, and investigators were unaware of the intervention patients received.
Bias in selection of the reported result	Low risk	Reported results for multiple outcome measurements which is clearly defined correspond to intended analyses, and it seems to be based on the protocol.

Ghaleiha 2014, Galantamine efficacy and tolerability as an augmentative therapy in autistic children: A randomized, double-blind, placebo-controlled trial

**Bias**	**Author's judgement**	**Support for judgement**
Bias arising from the randomization process	Low risk	Allocation sequence was adequately concealed. Baseline characteristics suggests no problem.
Bias due to deviations from intended interventions	Some concerns	Participants were unaware of the intervention. Appropriate analysis was not taken, but the impact does not seem substantial.
Bias due to missing outcome data	Low risk	17% (8 out of 48) participants were missed, and they took 'last-observation-carried-forward' method which could not correct the bias. Though, the missingness do not depend on true value.
Bias in measurement of the outcome	Low risk	Outcome measurement was consistent in both intervention groups, and investigators were unaware of the intervention patients received.
Bias in selection of the reported result	Low risk	Reported results for a single outcome measurement which is clearly defined correspond to intended analyses, and it seems to be based on the protocol.

Ghaleiha 2015, A pilot double-blind placebo-controlled trial of pioglitazone as adjunctive treatment to risperidone: Effects on aberrant behavior in children with autism

Bias	Author's judgement	Support for judgement
Bias arising from the randomization process	Low risk	Allocation sequence was adequately concealed. Baseline characteristics suggests no problem.
Bias due to deviations from intended interventions	Low risk	Participants were unaware of the intervention. Appropriate analysis as taken, thus no deviations from the intended interventions is expected.
Bias due to missing outcome data	Some concerns	9% (4 out of 44) participants were missed, and there was no information about the effort to account the bias. No information about whether the missingness depend on true value.
Bias in measurement of the outcome	Low risk	Outcome measurement was consistent in both intervention groups, and investigators were unaware of the intervention patients received.
Bias in selection of the reported result	Low risk	Reported results for a single outcome measurement which is clearly defined correspond to intended analyses, and it seems to be based on the protocol.

Ghaleiha 2016, Minocycline as Adjunctive Treatment to Risperidone in Children with Autistic Disorder: A Randomized, Double-Blind Placebo-Controlled Trial

**Bias**	**Author's judgement**	**Support for judgement**
Bias arising from the randomization process	Low risk	Allocation sequence seems to be concealed to participants. Baseline characteristics suggests no problem.
Bias due to deviations from intended interventions	Some concerns	Participants were aware of the intervention, but no deviations are expected. Appropriate analysis was taken.
Bias due to missing outcome data	Low risk	24% (15 out of 63) participants were missed, and there was no information about the effort to account the bias. Though, missingness do not seem to depend on true value.
Bias in measurement of the outcome	Some concerns	Outcome measurement was consistent in both intervention groups, but investigators were aware of the intervention patients received which could have influenced by knowledge of intervention.
Bias in selection of the reported result	Low risk	Reported results for multiple outcome measurements which is clearly defined correspond to intended analyses, and it seems to be based on the protocol.

Ghanizadeh 2013, A randomized double blind placebo controlled clinical trial of N-Acetylcysteine added to risperidone for treating autistic disorders

**Bias**	**Author's judgement**	**Support for judgement**
Bias arising from the randomization process	Low risk	Allocation sequence was adequately concealed. Baseline characteristics suggests no problem.
Bias due to deviations from intended interventions	Low risk	Participants were unaware of the intervention. Appropriate analysis (ITT analysis) was taken, thus no deviations from the intended interventions is expected.
Bias due to missing outcome data	Low risk	22.5% (9 out of 40) participants were missed, and they took 'last-observation-carried-forward' method which could not correct the bias. Though, the missingness do not depend on true value
Bias in measurement of the outcome	Low risk	Outcome measurement was consistent in both intervention groups, and investigators were unaware of the intervention patients received.
Bias in selection of the reported result	Low risk	Reported results for a single outcome measurement which is clearly defined correspond to intended analyses, and it seems to be based on the protocol.

Ginn 2017, Child-Directed Interaction Training for Young Children With Autism Spectrum Disorders: Parent and Child Outcomes

Bias	Author's judgement	Support for judgement
Bias arising from the randomization process	Low risk	Allocation sequence seems to be concealed to participants. Baseline characteristics suggests no problem.
Bias due to deviations from intended interventions	High risk	Participants were aware of the intervention, and deviation due to the trial context which no balanced between intervention groups has a potential to have a substantial impact.
Bias due to missing outcome data	Some concerns	23% (9 out of 39) participants were missed, and there was no information about the effort to account the bias. No information about whether the missingness depend on true value.
Bias in measurement of the outcome	Some concerns	Outcome measurement was consistent in both intervention groups, but investigators were aware of the intervention patients received which could have influenced by knowledge of intervention.
Bias in selection of the reported result	Low risk	Reported results for multiple outcome measurements which is clearly defined correspond to intended analyses, and it seems to be based on the protocol.

Hajizadeh-Zaker 2018, l-Carnosine As an Adjunctive Therapy to Risperidone in Children with Autistic Disorder: A Randomized, Double-Blind, Placebo-Controlled Trial

**Bias**	**Author's judgement**	**Support for judgement**
Bias arising from the randomization process	Low risk	Allocation sequence seems to be concealed to participants. Baseline characteristics suggests no problem.
Bias due to deviations from intended interventions	Some concerns	Participants were unaware of the intervention. Appropriate analysis was not taken, but the impact does not seem substantial.
Bias due to missing outcome data	Low risk	16% (8 out of 50) participants were missed, and there was no information about the effort to account the bias. Though, missingness do not seem to depend on true value.
Bias in measurement of the outcome	Low risk	Outcome measurement was consistent in both intervention groups, and investigators were unaware of the intervention patients received.
Bias in selection of the reported result	Low risk	Reported results for multiple outcome measurements which is clearly defined correspond to intended analyses, and it seems to be based on the protocol.

Handen 2015, Atomoxetine, Parent Training, and Their Combination in Children With Autism Spectrum Disorder and Attention-Deficit/Hyperactivity Disorder

**Bias**	**Author's judgement**	**Support for judgement**
Bias arising from the randomization process	Low risk	Allocation sequence seems to be concealed to participants. Baseline characteristics suggests no problem.
Bias due to deviations from intended interventions	Some concerns	Participants were aware of the intervention(parent training), but no deviations are expected. Appropriate analysis (ITT analysis) was taken.
Bias due to missing outcome data	Low risk	Outcome data were available for nearly all randomized participants.
Bias in measurement of the outcome	Low risk	Outcome measurement was consistent in intervention groups, and raters were unaware of the intervention patients received.
Bias in selection of the reported result	Low risk	Reported results for multiple outcome measurements which is clearly defined correspond to intended analyses, and it seems to be based on the protocol.

Hardan 2012, A randomized controlled pilot trial of oral N-acetylcysteine in children with autism

Bias	Author's judgement	Support for judgement
Bias arising from the randomization process	Low risk	Allocation sequence was adequately concealed. Baseline characteristics suggests no problem.
Bias due to deviations from intended interventions	Low risk	Participants were unaware of the intervention. Appropriate analysis was taken, thus no deviations from the intended interventions is expected.
Bias due to missing outcome data	Low risk	6.4% (4 out of 33) participants were missed, but there was no information about the effort to account the bias. Though the missingness do not depend on true value.
Bias in measurement of the outcome	Low risk	Outcome measurement was consistent in both intervention groups, and investigators were unaware of the intervention patients received.
Bias in selection of the reported result	Low risk	Reported results for multiple outcome measurements which is clearly defined correspond to intended analyses, and it seems to be based on the protocol.

Hasanzadeh 2012, A Double-Blind Placebo Controlled Trial of *Ginkgo biloba* Added to Risperidone in Patients with Autistic Disorders

**Bias**	**Author's judgement**	**Support for judgement**
Bias arising from the randomization process	Low risk	Allocation sequence seems to be concealed to participants. Baseline characteristics suggests no problem.
Bias due to deviations from intended interventions	Low risk	Participants were unaware of the intervention. Appropriate analysis was taken, thus no deviations from the intended interventions is expected.
Bias due to missing outcome data	Low risk	There is no missing data.
Bias in measurement of the outcome	Low risk	Outcome measurement was consistent in both intervention groups, and investigators were unaware of the intervention patients received.
Bias in selection of the reported result	Low risk	Reported results for multiple outcome measurements which is clearly defined correspond to intended analyses, and it seems to be based on the protocol.

Hellings 2005, A double-blind, placebo-controlled study of valproate for aggression in youth with pervasive developmental disorders

Bias	Author's judgement	Support for judgement
Bias arising from the randomization process	Some concerns	Allocation sequence seems to be concealed to participants, and no detailed information was presented on randomization method. Baseline characteristics suggests some problems(subject heterogeneity).
Bias due to deviations from intended interventions	Low risk	Participants were unaware of the drug assignment. Appropriate analysis was taken, thus no deviations from the intended interventions is expected.
Bias due to missing outcome data	Low risk	There was no missing data.
Bias in measurement of the outcome	Low risk	Outcome measurement was consistent in both intervention groups, and investigators were unaware of the intervention patients received.
Bias in selection of the reported result	Low risk	Reported results for multiple outcome measurement which is clearly defined correspond to intended analyses, and it seems to be based on the protocol.

Hendouei 2020, Resveratrol as adjunctive therapy in treatment of irritability in children with autism: A double-blind and placebo-controlled randomized trial

**Bias**	**Author’s judgement**	**Support for judgement**
Bias arising from the randomization process	Low risk	Allocation sequence adequately concealed to participants. Baseline characteristics suggests no problem.
Bias due to deviations from intended interventions	Low risk	Participants were unaware of the intervention. Appropriate analysis was taken, thus no deviations from the intended interventions is expected.
Bias due to missing outcome data	Low risk	11% (8 out of 70) participants were missed, and there was no information about the effort to account the bias. Though, missingness do not seem to depend on true value.
Bias in measurement of the outcome	Low risk	Outcome measurement was consistent in both intervention groups, and investigators were unaware of the intervention patients received.
Bias in selection of the reported result	Low risk	Reported results for multiple outcome measurements which is clearly defined correspond to intended analyses, and it seems to be based on the protocol.

Hollander 2010, Divalproex sodium vs placebo for the treatment of irritability in children and adolescents with autism spectrum disorders

Bias	Author's judgement	Support for judgement
Bias arising from the randomization process	Low risk	Allocation sequence seems to be concealed to participants, and no detailed information was presented on randomization method. Baseline characteristics suggests no problem.
Bias due to deviations from intended interventions	Low risk	Participants were unaware of the intervention. Appropriate analysis (ITT analysis) was taken, thus no deviations from the intended interventions is expected.
Bias due to missing outcome data	Low risk	There was no missing data.
Bias in measurement of the outcome	Low risk	Outcome measurement was consistent in both intervention groups, and investigators were unaware of the intervention patients received.
Bias in selection of the reported result	Low risk	Reported results for multiple outcome measurements which is clearly defined correspond to intended analyses, and it seems to be based on the protocol.

Hollander 2022, Balovaptan vs placebo for social communication in childhood autism spectrum disorder a randomized clinical trial

**Bias**	**Author's judgement**	**Support for judgement**
Bias arising from the randomization process	Low risk	Allocation sequence adequately concealed to participants. Baseline characteristics suggests no problem.
Bias due to deviations from intended interventions	High risk	Participants were unaware of the intervention. Appropriate analysis was not taken, and the might have substantial impact because the number of excluded participants is not small.
Bias due to missing outcome data	Low risk	46% (113 out of 248) participants were missed, but there was an effort to account the bias.
Bias in measurement of the outcome	Low risk	Outcome measurement was consistent in both intervention groups, and investigators were unaware of the intervention patients received.
Bias in selection of the reported result	Low risk	Reported results for multiple outcome measurements which is clearly defined correspond to intended analyses, and it seems to be based on the protocol.

Ichikawa 2017, Aripiprazole in the Treatment of Irritability in Children and Adolescents with Autism Spectrum Disorder in Japan: A Randomized, Double-blind, Placebo-controlled Study

Bias	Author's judgement	Support for judgement
Bias arising from the randomization process	Low risk	Allocation sequence seems to be concealed to participants, and no detailed information was presented on randomization method. Baseline characteristics suggests no problem.
Bias due to deviations from intended interventions	Low risk	Participants were unaware of the drug assignment. Appropriate analysis was taken, thus no deviations from the intended interventions is expected.
Bias due to missing outcome data	Low risk	Outcome data were available for nearly all randomized participants.
Bias in measurement of the outcome	Low risk	Outcome measurement was consistent in both intervention groups, and investigators were unaware of the intervention patients received.
Bias in selection of the reported result	Low risk	Reported results for a multiple outcome measurement which is clearly defined correspond to intended analyses, and it seems to be based on the protocol.

Kent 2013, Risperidone dosing in children and adolescents with autistic disorder: a double-blind, placebo-controlled study

**Bias**	**Author's judgement**	**Support for judgement**
Bias arising from the randomization process	Low risk	Allocation sequence was adequately concealed. Baseline characteristics suggests no problem.
Bias due to deviations from intended interventions	Low risk	Participants were unaware of the intervention. Appropriate analysis (ITT analysis) was taken, thus no deviations from the intended interventions is expected.
Bias due to missing outcome data	Low risk	There was no missing data.
Bias in measurement of the outcome	Low risk	Outcome measurement was consistent in both intervention groups, and investigators were unaware of the intervention patients received.
Bias in selection of the reported result	Low risk	Reported results for multiple outcome measurements which is clearly defined correspond to intended analyses, and it seems to be based on the protocol.

Kerley 2017, Lack of effect of vitamin D3 supplementation in autism: a 20-week, placebo-controlled RCT

Bias	Author's judgement	Support for judgement
Bias arising from the randomization process	Low risk	Allocation sequence seems to be concealed to participants. Baseline characteristics suggests no problem.
Bias due to deviations from intended interventions	Low risk	Participants were unaware of the intervention. Appropriate analysis was taken, thus no deviations from the intended interventions is expected.
Bias due to missing outcome data	Low risk	9.5% (4 out of 42) participants were missed, and there was no information about the effort to account the bias. Though, missingness do not seem to depend on true value.
Bias in measurement of the outcome	Low risk	Outcome measurement was consistent in intervention groups, and raters were unaware of the intervention patients received.
Bias in selection of the reported result	Low risk	Reported results for multiple outcome measurements which is clearly defined correspond to intended analyses, and it seems to be based on the protocol.

Khalaj 2018, Palmitoylethanolamide as adjunctive therapy for autism: Efficacy and safety results from a randomized controlled trial

**Bias**	**Author's judgement**	**Support for judgement**
Bias arising from the randomization process	Low risk	Allocation sequence is adequately concealed to participants. Baseline characteristics suggests no problem.
Bias due to deviations from intended interventions	Low risk	Participants were unaware of the intervention. Appropriate analysis (ITT analysis) was taken, thus no deviations from the intended interventions is expected.
Bias due to missing outcome data	Low risk	11% (8 out of 70) participants were missed, but there was an effort to account the bias.
Bias in measurement of the outcome	Low risk	Outcome measurement was consistent in both intervention groups, and investigators were unaware of the intervention patients received.
Bias in selection of the reported result	Low risk	Reported results for a single outcome measurement which is clearly defined correspond to intended analyses, and it seems to be based on the protocol.

King 2001, Double-blind, placebo-controlled study of amantadine hydrochloride in the treatment of children with autistic disorder

Bias	Author's judgement	Support for judgement
Bias arising from the randomization process	Low risk	Allocation sequence seems to be concealed to participants, and no detailed information was presented on randomization method. Baseline characteristics suggests no problem.
Bias due to deviations from intended interventions	Low risk	Participants were unaware of the drug assignment. no deviation due to the trial context was occurred.
Bias due to missing outcome data	Low risk	There was no missing data.
Bias in measurement of the outcome	Low risk	Outcome measurement was consistent in both intervention groups, and investigators were unaware of the intervention patients received.
Bias in selection of the reported result	Some concerns	Results of multiple outcome measurements were analyzed in pre-specified plan but the result being assessed is likely to have been selected.

Kong 2021, Probiotic and Oxytocin Combination Therapy in Patients with Autism Spectrum Disorder: A Randomized, Double-Blinded, Placebo-Controlled Pilot Trial

**Bias**	**Author's judgement**	**Support for judgement**
Bias arising from the randomization process	Low risk	Allocation sequence was adequately concealed. Baseline characteristics suggests no problem.
Bias due to deviations from intended interventions	Low risk	Participants were unaware of the drug assignment. Appropriate analysis (ITT analysis) was taken, thus no deviations from the intended interventions is expected.
Bias due to missing outcome data	Low risk	23% (8 out of 35) participants were missed, and there was no information about the effort to account the bias. Though, missingness do not seem to depend on true value.
Bias in measurement of the outcome	Low risk	Outcome measurement was consistent in both intervention groups, and investigators were unaware of the intervention patients received.
Bias in selection of the reported result	Low risk	Reported results for a multiple outcome measurement which is clearly defined correspond to intended analyses, and it seems to be based on the protocol.

Loebel 2016, Lurasidone for the Treatment of Irritability Associated with Autistic Disorder

**Bias**	**Author's judgement**	**Support for judgement**
Bias arising from the randomization process	Low risk	Allocation sequence seems to be concealed to participants, and no detailed information was presented on randomization method. Baseline characteristics suggests no problem.
Bias due to deviations from intended interventions	Low risk	Participants were unaware of the intervention. Appropriate analysis (ITT analysis) was taken, thus no deviations from the intended interventions is expected.
Bias due to missing outcome data	Some concerns	15% (22 out of 150) participants were missed, and they took 'last-observation-carried-forward' method which could not correct the bias. Some missingness could depend on true value.
Bias in measurement of the outcome	Low risk	Outcome measurement was consistent in both intervention groups, and investigators were unaware of the intervention patients received.
Bias in selection of the reported result	Low risk	Reported results for a single outcome measurement which is clearly defined correspond to intended analyses, and it seems to be based on the protocol.

Mahdavinasab 2019, Baclofen as an adjuvant therapy for autism: a randomized, double-blind, placebo-controlled trial

**Bias**	**Author's judgement**	**Support for judgement**
Bias arising from the randomization process	Low risk	Allocation sequence seems to be concealed to participants. Baseline characteristics suggests no problem.
Bias due to deviations from intended interventions	Low risk	Participants were unaware of the intervention. Appropriate analysis was taken, thus no deviations from the intended interventions is expected.
Bias due to missing outcome data	Low risk	9.4% (6 out of 64) participants were missed, and there was no information about the effort to account the bias. Though, missingness do not seem to depend on true value.
Bias in measurement of the outcome	Low risk	Outcome measurement was consistent in both intervention groups, and investigators were unaware of the intervention patients received.
Bias in selection of the reported result	Low risk	Reported results for a single outcome measurement which is clearly defined correspond to intended analyses, and it seems to be based on the protocol.

Malek 2020, Prednisolone as Adjunctive Treatment to Risperidone in Children With Regressive Type of Autism Spectrum Disorder: A Randomized, Placebo-Controlled Trial

**Bias**	**Author's judgement**	**Support for judgement**
Bias arising from the randomization process	Low risk	Allocation sequence seems to be concealed to participants. Baseline characteristics suggests no problem.
Bias due to deviations from intended interventions	Low risk	Participants were unaware of the intervention. Appropriate analysis was taken, thus no deviations from the intended interventions is expected.
Bias due to missing outcome data	Low risk	30% (11 out of 37) participants were missed, and there was no information about the effort to account the bias. Though, missingness do not seem to depend on true value.
Bias in measurement of the outcome	Low risk	Outcome measurement was consistent in both intervention groups, and investigators were unaware of the intervention patients received.
Bias in selection of the reported result	Low risk	Reported results for multiple outcome measurements which is clearly defined correspond to intended analyses, and it seems to be based on the protocol.

Marcus 2009, A placebo-controlled, fixed-dose study of aripiprazole in children and adolescents with irritability associated with autistic disorder

Bias	Author's judgement	Support for judgement
Bias arising from the randomization process	Low risk	Allocation sequence seems to be concealed to participants. Baseline characteristics suggests no problem.
Bias due to deviations from intended interventions	Low risk	Participants were unaware of the intervention. Appropriate analysis was taken, thus no deviations from the intended interventions is expected.
Bias due to missing outcome data	Low risk	Outcome data were available for nearly all randomized participants.
Bias in measurement of the outcome	Low risk	Outcome measurement was consistent in both intervention groups, and investigators were unaware of the intervention patients received.
Bias in selection of the reported result	Low risk	Reported results for multiple outcome measurements which is clearly defined correspond to intended analyses, and it seems to be based on the protocol.

Mazahery 2019, A randomised controlled trial of vitamin D and omega-3 long chain polyunsaturated fatty acids in the treatment of irritability and hyperactivity among children with autism spectrum disorder

Bias	Author's judgement	Support for judgement
Bias arising from the randomization process	Low risk	Allocation sequence seems to be concealed to participants and no detailed information was presented on randomization method. Baseline characteristics suggests no problem.
Bias due to deviations from intended interventions	Low risk	Participants were unaware of the intervention. Appropriate analysis was taken, thus no deviations from the intended interventions is expected.
Bias due to missing outcome data	Some concerns	34% (38 out of 111) participants were missed, and there was no information about the effort to account the bias. No information whether the missingness depend on true value.
Bias in measurement of the outcome	Low risk	Outcome measurement was consistent in both intervention groups, and investigators were unaware of the intervention patients received.
Bias in selection of the reported result	Low risk	Reported results for multiple outcome measurements which is clearly defined correspond to intended analyses, and it seems to be based on the protocol.

McCracken 2002, Risperidone in children with autism and serious behavioral problems

Bias	Author’s judgement	Support for judgement
Bias arising from the randomization process	Low risk	Allocation sequence seems to be concealed to participants, and no detailed information was presented on randomization method. Baseline characteristics suggests no problem.
Bias due to deviations from intended interventions	Low risk	Participants were unaware of the drug assignment. Appropriate analysis (ITT analysis) was taken, thus no deviations from the intended interventions is expected.
Bias due to missing outcome data	Low risk	There was no missing data.
Bias in measurement of the outcome	Low risk	Outcome measurement was consistent in both intervention groups, and investigators were unaware of the intervention patients received.
Bias in selection of the reported result	Low risk	Reported results for multiple outcome measurements which is clearly defined correspond to intended analyses, and it seems to be based on the protocol.

McDougle 1998, A double-blind, placebo-controlled study of risperidone in adults with autistic disorder and other pervasive developmental disorders

Bias	Author's judgement	Support for judgement
Bias arising from the randomization process	Low risk	Allocation sequence was adequately concealed. Baseline characteristics suggest no problem.
Bias due to deviations from intended interventions	Low risk	Participants were unaware of the drug assignment. no deviation due to the trial context was occurred. Appropriate analysis (ITT analysis) was used.
Bias due to missing outcome data	High risk	22.6% (7 out of 31) participants were missed, no effort was made to correct the potential bias. There is no information whether the missingness in the outcome depend on its true value.
Bias in measurement of the outcome	Low risk	Outcome measurement was consistent in both intervention groups, and investigators were unaware of the intervention patients received.
Bias in selection of the reported result	Low risk	Reported results for a single outcome measurement which is clearly defined correspond to intended analyses, and it seems to be based on the protocol.

Moazen-Zadeh 2018, Simvastatin as an Adjunctive Therapy to Risperidone in Treatment of Autism: A Randomized, Double-Blind, Placebo-Controlled Clinical Trial

Bias	Author's judgement	Support for judgement
Bias arising from the randomization process	Low risk	Allocation sequence was adequately concealed. Baseline characteristics suggests no problem.
Bias due to deviations from intended interventions	Low risk	Participants were unaware of the intervention. Appropriate analysis was taken, thus no deviations from the intended interventions is expected.
Bias due to missing outcome data	Low risk	5.7% (4 out of 70) participants were missed, and there was no information about the effort to account the bias. Though, missingness do not seem to depend on true value.
Bias in measurement of the outcome	Low risk	Outcome measurement was consistent in both intervention groups, and investigators were unaware of the intervention patients received.
Bias in selection of the reported result	Low risk	Reported results for a single outcome measurement which is clearly defined correspond to intended analyses, and it seems to be based on the protocol.

Mohammadi 2013, Double-Blind, Placebo-Controlled Trial of Risperidone Plus Amantadine in Children With Autism: A 10-Week Randomized Study

Bias	Author's judgement	Support for judgement
Bias arising from the randomization process	Low risk	Allocation sequence was adequately concealed. Baseline characteristics suggests no problem.
Bias due to deviations from intended interventions	Low risk	Participants were unaware of the intervention. Appropriate analysis (ITT analysis) was taken, thus no deviations from the intended interventions is expected.
Bias due to missing outcome data	Low risk	Outcome data were available for nearly all randomized participants.
Bias in measurement of the outcome	Low risk	Outcome measurement was consistent in both intervention groups, and investigators were unaware of the intervention patients received.
Bias in selection of the reported result	Low risk	Reported results for a single outcome measurement which is clearly defined correspond to intended analyses, and it seems to be based on the protocol.

Momtazmanesh 2020, Sulforaphane as an adjunctive treatment for irritability in children with autism spectrum disorder: A randomized, double-blind, placebo-controlled clinical trial

**Bias**	**Author's judgement**	**Support for judgement**
Bias arising from the randomization process	Low risk	Allocation sequence seems to be concealed to participants. Baseline characteristics suggests no problem.
Bias due to deviations from intended interventions	Low risk	Participants were unaware of the intervention. Appropriate analysis was taken, thus no deviations from the intended interventions is expected.
Bias due to missing outcome data	Low risk	12% (8 out of 68) participants were missed, and there was no information about the effort to account the bias. Though, missingness do not seem to depend on true value.
Bias in measurement of the outcome	Low risk	Outcome measurement was consistent in both intervention groups, and investigators were unaware of the intervention patients received.
Bias in selection of the reported result	Low risk	Reported results for multiple outcome measurements which is clearly defined correspond to intended analyses, and it seems to be based on the protocol.

Nikoo 2015, N-acetylcysteine as an adjunctive therapy to risperidone for treatment of irritability in autism: a randomized, double-blind, placebo-controlled clinical trial of efficacy and safety

**Bias**	**Author's judgement**	**Support for judgement**
Bias arising from the randomization process	Low risk	Allocation sequence was adequately concealed. Baseline characteristics suggests no problem.
Bias due to deviations from intended interventions	Some concerns	Participants were unaware of the intervention. Appropriate analysis was not taken, but the impact does not seem substantial.
Bias due to missing outcome data	Some concerns	20% (10 out of 50) participants were missed, and there was no information about the effort to account the bias. No information about whether the missingness depend on true value.
Bias in measurement of the outcome	Low risk	Outcome measurement was consistent in both intervention groups, and investigators were unaware of the intervention patients received.
Bias in selection of the reported result	Low risk	Reported results for a single outcome measurement which is clearly defined correspond to intended analyses, and it seems to be based on the protocol.

Owen 2009, Aripiprazole in the treatment of irritability in children and adolescents with autistic disorder

Bias	Author's judgement	Support for judgement
Bias arising from the randomization process	Low risk	Allocation sequence seems to be concealed to participants. Baseline characteristics suggests no problem.
Bias due to deviations from intended interventions	Low risk	Participants were unaware of the intervention. Appropriate analysis was taken, thus no deviations from the intended interventions is expected.
Bias due to missing outcome data	Low risk	Outcome data were available for nearly all randomized participants.
Bias in measurement of the outcome	Low risk	Outcome measurement was consistent in both intervention groups, and investigators were unaware of the intervention patients received.
Bias in selection of the reported result	Low risk	Reported results for multiple outcome measurements which is clearly defined correspond to intended analyses, and it seems to be based on the protocol.

Pandina 2007, Risperidone improves behavioral symptoms in children with autism in a randomized, double-blind, placebo-controlled trial

**Bias**	**Author's judgement**	**Support for judgement**
Bias arising from the randomization process	Low risk	Allocation sequence seems to be concealed to participants, and no detailed information was presented on randomization method. Baseline characteristics suggests no problem.
Bias due to deviations from intended interventions	Some concerns	Participants were unaware of the drug assignment, but the analysis seems inappropriate.
Bias due to missing outcome data	Low risk	5.5% (3 out of 55) participants were missed, but there was an effort to account the bias.
Bias in measurement of the outcome	Low risk	Outcome measurement was consistent in both intervention groups, and investigators were unaware of the intervention patients received.
Bias in selection of the reported result	Low risk	Reported results for multiple outcome measurements which is clearly defined correspond to intended analyses, and it seems to be based on the protocol.

Rezaei 2010, Double-blind, placebo-controlled trial of risperidone plus topiramate in children with autistic disorder

Bias	Author's judgement	Support for judgement
Bias arising from the randomization process	Low risk	Allocation sequence was adequately concealed. Baseline characteristics suggests no problem.
Bias due to deviations from intended interventions	Low risk	Participants were unaware of the intervention. Appropriate analysis was taken, thus no deviations from the intended interventions is expected.
Bias due to missing outcome data	Low risk	There was no missing data.
Bias in measurement of the outcome	Low risk	Outcome measurement was consistent in both intervention groups, and investigators were unaware of the intervention patients received.
Bias in selection of the reported result	Low risk	Reported results for a single outcome measurement which is clearly defined correspond to intended analyses, and it seems to be based on the protocol.

Rezaei 2018, Effects of Combining Medication and Pivotal Response Treatment on Aberrant Behavior in Children with Autism Spectrum Disorder

**Bias**	**Author's judgement**	**Support for judgement**
Bias arising from the randomization process	Low risk	Allocation sequence seems to be concealed to participants, and no detailed information was presented on randomization method. Baseline characteristics suggests no problem.
Bias due to deviations from intended interventions	High risk	Participants were aware of the intervention, and deviation due to the trial context which no balanced between intervention groups has a potential to have an impact on the result since the number of total participants was small.
Bias due to missing outcome data	Low risk	5.6% (2 out of 36) participants were missed, and there was no information about the effort to account the bias. Though, missingness do not seem to depend on true value.
Bias in measurement of the outcome	Some concerns	Outcome measurement was consistent in both intervention groups, but investigators were aware of the intervention patients received which could have influenced by knowledge of intervention.
Bias in selection of the reported result	Low risk	Reported results for multiple outcome measurements which is clearly defined correspond to intended analyses, and it seems to be based on the protocol.

Rossigno 2009, Hyperbaric treatment for children with autism: a multicenter, randomized, double-blind, controlled trial

Bias	Author's judgement	Support for judgement
Bias arising from the randomization process	Low risk	Allocation sequence seems to be concealed to participants. Baseline characteristics suggests no problem.
Bias due to deviations from intended interventions	Low risk	Participants were unaware of the intervention. Appropriate analysis (ITT analysis) was taken, thus no deviations from the intended interventions is expected.
Bias due to missing outcome data	Low risk	9.7% (6 out of 62) participants were missed, but there was an effort to account the bias.
Bias in measurement of the outcome	Low risk	Outcome measurement was consistent in both intervention groups, and investigators were unaware of the intervention patients received.
Bias in selection of the reported result	Low risk	Reported results for multiple outcome measurements which is clearly defined correspond to intended analyses, and it seems to be based on the protocol.

Scahill 2015, Extended-Release Guanfacine for Hyperactivity in Children With Autism Spectrum Disorder

**Bias**	**Author's judgement**	**Support for judgement**
Bias arising from the randomization process	Low risk	Allocation sequence seems to be concealed to participants. Baseline characteristics suggests no problem.
Bias due to deviations from intended interventions	Low risk	Participants were unaware of the intervention. Appropriate analysis was taken, thus no deviations from the intended interventions is expected.
Bias due to missing outcome data	Low risk	Outcome data were available for all randomized participants.
Bias in measurement of the outcome	Low risk	Outcome measurement was consistent in both intervention groups, and investigators were unaware of the intervention patients received.
Bias in selection of the reported result	Low risk	Reported results for multiple outcome measurements which is clearly defined correspond to intended analyses, and it seems to be based on the protocol.

Shea 2004, Risperidone in the treatment of disruptive behavioral symptoms in children with autistic and other pervasive developmental disorders

**Bias**	**Author's judgement**	**Support for judgement**
Bias arising from the randomization process	Some concerns	Allocation sequence seems to be concealed to participants, and no detailed information was presented on randomization method. Baseline characteristics suggests a problem.
Bias due to deviations from intended interventions	Low risk	Participants were unaware of the drug assignment. Appropriate analysis (ITT analysis) was taken, thus no deviations from the intended interventions is expected.
Bias due to missing outcome data	Low risk	Outcome data were available for nearly all randomized participants.
Bias in measurement of the outcome	Low risk	Outcome measurement was consistent in both intervention groups, and investigators were unaware of the intervention patients received.
Bias in selection of the reported result	Low risk	Reported results for single outcome measurement which is clearly defined correspond to intended analyses, and it seems to be based on the protocol.

Singh 2014, Sulforaphane treatment of autism spectrum disorder (ASD)

**Bias**	**Author's judgement**	**Support for judgement**
Bias arising from the randomization process	Low risk	Allocation sequence seems to be concealed to participants, and no detailed information was presented on randomization method. Baseline characteristics suggests no problem.
Bias due to deviations from intended interventions	Low risk	Participants were unaware of the intervention. Appropriate analysis was taken, thus no deviations from the intended interventions is expected.
Bias due to missing outcome data	Low risk	9% (4 out of 44) participants were missed, but there was an effort to account the bias.
Bias in measurement of the outcome	Low risk	Outcome measurement was consistent in both intervention groups, and investigators were unaware of the intervention patients received.
Bias in selection of the reported result	Low risk	Reported results for multiple outcome measurements which is clearly defined correspond to intended analyses, and it seems to be based on the protocol.

Sofronoff 2004, Parent management training and Asperger syndrome: a randomized controlled trial to evaluate a parent based intervention

Bias	Author’s judgement	Support for judgement
Bias arising from the randomization process	Low risk	Allocation sequence seems to be concealed to participants, and no detailed information was presented on randomization method. Baseline characteristics suggests no problem.
Bias due to deviations from intended interventions	High risk	Participants were aware of the intervention, and deviation due to the trial context which no balanced between intervention groups has a potential to have an impact on the result since the number of total participants was small.
Bias due to missing outcome data	Low risk	There was no missing data.
Bias in measurement of the outcome	Some concerns	Outcome measurement was consistent in intervention groups. But investigators were aware of the intervention, and could have been influenced by knowledge of intervention received, though no reason .
Bias in selection of the reported result	Low risk	Reported results for multiple outcome measurements which is clearly defined correspond to intended analyses, and it seems to be based on the protocol.

Sprengers 2021, Bumetanide for Core Symptoms of Autism Spectrum Disorder (BAMBI): A Single Center, Double-blinded, Participant-Randomized, Placebo-Controlled, Phase-2 Superiority Trial

**Bias**	**Author's judgement**	**Support for judgement**
Bias arising from the randomization process	Low risk	Allocation sequence was adequately concealed. Baseline characteristics suggests no problem.
Bias due to deviations from intended interventions	Low risk	Participants were unaware of the drug assignment. Appropriate analysis (modified ITT analysis) was taken, thus no deviations from the intended interventions is expected.
Bias due to missing outcome data	Some concerns	11% (10 out of 92) participants were missed, and there was no information about the effort to account the bias. Some missingness could depend on true value.
Bias in measurement of the outcome	Low risk	Outcome measurement was consistent in both intervention groups, and investigators were unaware of the intervention patients received.
Bias in selection of the reported result	Low risk	Reported results for a multiple outcome measurement which is clearly defined correspond to intended analyses, and it seems to be based on the protocol.

Veenstra-Vanderweele 2017, Arbaclofen in Children and Adolescents with Autism Spectrum Disorder: A Randomized, Controlled, Phase 2 Trial

**Bias**	**Author's judgement**	**Support for judgement**
Bias arising from the randomization process	Low risk	Allocation sequence seems to be concealed to participants, and no detailed information was presented on randomization method. Baseline characteristics suggests no problem.
Bias due to deviations from intended interventions	Low risk	Participants were unaware of the drug assignment. Appropriate analysis (ITT analysis) was taken, thus no deviations from the intended interventions is expected.
Bias due to missing outcome data	Low risk	13% (20 out of 150) participants were missed, but there was an effort to account the bias.
Bias in measurement of the outcome	Low risk	Outcome measurement was consistent in both intervention groups, and investigators were unaware of the intervention patients received.
Bias in selection of the reported result	Low risk	Reported results for a multiple outcome measurement which is clearly defined correspond to intended analyses, and it seems to be based on the protocol.

Wasserman 2006, Levetiracetam versus placebo in childhood and adolescent autism: a double-blind placebo-controlled study

Bias	Author's judgement	Support for judgement
Bias arising from the randomization process	Low risk	Allocation sequence seems to be concealed to participants, and no detailed information was presented on randomization method. Baseline characteristics suggests no problem.
Bias due to deviations from intended interventions	Low risk	Participants were unaware of the drug assignment. Appropriate analysis (ITT analysis) was taken, thus no deviations from the intended interventions is expected.
Bias due to missing outcome data	Low risk	There was no missing data.
Bias in measurement of the outcome	Low risk	Outcome measurement was consistent in both intervention groups, and investigators were unaware of the intervention patients received.
Bias in selection of the reported result	Low risk	Reported results for a multiple outcome measurement which is clearly defined correspond to intended analyses, and it seems to be based on the protocol.

Whittingham 2009, Stepping Stones Triple P: an RCT of a parenting program with parents of a child diagnosed with an autism spectrum disorder

**Bias**	**Author's judgement**	**Support for judgement**
Bias arising from the randomization process	Low risk	Allocation sequence seems to be concealed to participants, and no detailed information was presented on randomization method. Baseline characteristics suggests no problem.
Bias due to deviations from intended interventions	High risk	Participants were aware of the intervention, and deviation due to the trial context which no balanced between intervention groups has a potential to have a substantial impact.
Bias due to missing outcome data	Low risk	There was no missing data.
Bias in measurement of the outcome	Some concerns	Outcome measurement was consistent in both intervention groups, but investigators were aware of the intervention patients received which could have influenced by knowledge of intervention.
Bias in selection of the reported result	Low risk	Reported results for multiple outcome measurements which is clearly defined correspond to intended analyses, and it seems to be based on the protocol.

Wink 2016, A randomized placebo-controlled pilot study of N-acetylcysteine in youth with autism spectrum disorder

Bias	Author's judgement	Support for judgement
Bias arising from the randomization process	Low risk	Allocation sequence was adequately concealed. Baseline characteristics suggests no problem.
Bias due to deviations from intended interventions	Low risk	Participants were unaware of the intervention. Appropriate analysis was taken, thus no deviations from the intended interventions is expected.
Bias due to missing outcome data	Some concerns	19% (6 out of 31) participants were missed, and there was no information about the effort to account the bias. Some missingness could depend on true value.
Bias in measurement of the outcome	Low risk	Outcome measurement was consistent in both intervention groups, and investigators were unaware of the intervention patients received.
Bias in selection of the reported result	Low risk	Reported results for multiple outcome measurements which is clearly defined correspond to intended analyses, and it seems to be based on the protocol.

Wong 2010, Randomized controlled trial of electro-acupuncture for autism spectrum disorder

**Bias**	**Author's judgement**	**Support for judgement**
Bias arising from the randomization process	Low risk	Allocation sequence was adequately concealed. Baseline characteristics suggests no problem.
Bias due to deviations from intended interventions	Low risk	Participants were unaware of the intervention. Appropriate analysis (ITT analysis) was taken, thus no deviations from the intended interventions is expected.
Bias due to missing outcome data	Low risk	6.8% (4 out of 59) participants were missed, but there was no information about the effort to account the bias. Though the missingness do not depend on true value.
Bias in measurement of the outcome	Low risk	Outcome measurement was consistent in both intervention groups, and investigators were unaware of the intervention patients received.
Bias in selection of the reported result	Low risk	Reported results for multiple outcome measurements which is clearly defined correspond to intended analyses, and it seems to be based on the protocol.

Yui 2012, Effects of large doses of arachidonic acid added to docosahexaenoic acid on social impairment in individuals with autism spectrum disorders: a double-blind, placebo-controlled, randomized trial

**Bias**	**Author's judgement**	**Support for judgement**
Bias arising from the randomization process	Low risk	Allocation sequence seems to be concealed to participants. Baseline characteristics suggests no problem.
Bias due to deviations from intended interventions	Low risk	Participants were unaware of the intervention. Appropriate analysis was taken, thus no deviations from the intended interventions is expected.
Bias due to missing outcome data	Low risk	There was no missing data.
Bias in measurement of the outcome	Low risk	Outcome measurement was consistent in both intervention groups, and investigators were unaware of the intervention patients received.
Bias in selection of the reported result	Low risk	Reported results for multiple outcome measurements which is clearly defined correspond to intended analyses, and it seems to be based on the protocol.

Zand 2018, A Pilot of a Brief Positive Parenting Program on Children Newly Diagnosed with Autism Spectrum Disorder

**Bias**	**Author's judgement**	**Support for judgement**
Bias arising from the randomization process	Low risk	Allocation sequence seems to be concealed to participants. Baseline characteristics suggests no problem.
Bias due to deviations from intended interventions	High risk	Participants were aware of the intervention, and deviation due to the trial context which no balanced between intervention groups has a potential to have a substantial impact.
Bias due to missing outcome data	Low risk	19% (5 out of 26) participants were missed, and there was no information about the effort to account the bias. Though, missingness do not seem to depend on true value.
Bias in measurement of the outcome	Some concerns	Outcome measurement was consistent in both intervention groups, but investigators were aware of the intervention patients received which could have influenced by knowledge of intervention.
Bias in selection of the reported result	Low risk	Reported results for multiple outcome measurements which is clearly defined correspond to intended analyses, and it seems to be based on the protocol.
